# Characterization, expression profiling, and functional analysis of a *Populus trichocarpa* defensin gene and its potential as an anti-*Agrobacterium* rooting medium additive

**DOI:** 10.1038/s41598-019-51762-0

**Published:** 2019-10-25

**Authors:** Hui Wei, Ali Movahedi, Chen Xu, Weibo Sun, Lingling Li, Dawei Li, Qiang Zhuge

**Affiliations:** 1 Co-Innovation Center for Sustainable Forestry in Southern China, Key Laboratory of Forest Genetics & Biotechnology, Ministry of Education, College of Biology and the Environment, Nanjing Forestry University, Nanjing, 210037 China; 2 Jiangsu Provincial Key Construction Laboratory of Special Biomass Resource Utilization, Nanjing Xiaozhuang University, Nanjing, 211171 China

**Keywords:** Plant molecular biology, Biotic

## Abstract

The diverse antimicrobial properties of defensins have attracted wide scientific interest in recent years. Also, antimicrobial peptides (AMPs), including cecropins, histatins, defensins, and cathelicidins, have recently become an antimicrobial research hotspot for their broad-spectrum antibacterial and antifungal activities. In addition, defensins play important roles in plant growth, development, and physiological metabolism, and demonstrate tissue specificity and regulation in response to pathogen attack or abiotic stress. In this study, we performed molecular cloning, characterization, expression profiling, and functional analysis of a defensin from *Populus trichocarpa*. The PtDef protein was highly expressed in the prokaryotic *Escherichia coli* system as a fusion protein (TrxA–PtDef). The purified protein exhibited strong antibacterial and antifungal functions. We then applied PtDef to rooting culture medium as an alternative exogenous additive to cefotaxime. *PtDef* expression levels increased significantly following both biotic and abiotic treatment. The degree of leaf damage observed in wild-type (WT) and transgenic poplars indicates that transgenic poplars that overexpress the *PtDef* gene gain enhanced disease resistance to *Septotis populiperda*. To further study the salicylic acid (SA) and jasmonic acid (JA) signal transduction pathways, SA- and JA-related and pathogenesis-related genes were analyzed using quantitative reverse-transcription polymerase chain reaction; there were significant differences in these pathways between transgenic and WT poplars. The defensin from *Populus trichocarpa* showed significant activity of anti-bacteria and anti-fungi. According to the results of qRT-PCR and physiological relevant indicators, the applied PtDef to rooting culture medium was chosen as an alternative exogenous additive to cefotaxime. Overexpressing the *PtDef* gene in poplar improve the disease resistance to *Septotis populiperda*.

## Introduction

Antimicrobial peptides (AMPs), including cecropins, histatins, defensins, and cathelicidins, have recently become an antimicrobial research hotspot for their broad-spectrum antibacterial and antifungal activities^1,2^. Studies have shown that cecropins isolated from silkworm moth hemolymph are positively charged peptides with a helix–bend–helix structure, which exhibit a broad spectrum of activity against both gram-negative and gram-positive bacteria^3,4^. Histatins isolated from primate saliva have been demonstrated to have antifungal activity^5^. Cathelicidin with a C-terminal peptide has the ability to resist group A streptococcus, and cathelicidin production appears to be mainly restricted to mammals^6,7^. Defensins have been isolated from a wide range of organisms^8–11^, and different plant defensins have shown low amino acid sequence similarities, except in conservative cysteine (Cys) residues^10–13^. Despite the large variability among amino acid sequences, many plant defensins exhibit similarities in three-dimensional (3D) structure, including three-stranded reverse-parallel β-sheets and an α-helix bound to β-sheets^13^. Defensins exhibit antimicrobial activity against fungi, gram-positive bacteria, and gram-negative bacteria; however, their activity is stronger against fungi than against bacteria^14–17^. Besides antimicrobial activity, defensins have also been shown to exhibit anti-human immunodeficiency virus (HIV) effects^18^. Defensin binds to viral coat proteins, causing the viruses to lose their biological activity. Human and mouse defensins can significantly inhibit or even kill enveloped viruses, herpes viruses, and the vesicular stomatitis virus^19^. Defensins have been shown to influence antitumor cell activity and may play an important role in tumor immunity^20^. Defensins also enhance acquired immunity by activating cellular and humoral immunity to kill and eliminate microorganisms^21^ through the attraction of immature dendritic cells and memory cells to inflammatory sites. Defensins can aggregate T cells and monocytes on the mucosal surface. At low concentrations, defensins do not kill microorganisms, but can play an immune surveillance role^22^. Plant defensins were discovered much later than mammalian defensins; however, after more than a decade of research, it has been confirmed that plant defensins are present in all plants, within various organs and tissues. Defensins play important roles in plant growth, development, and physiological metabolism, and demonstrate tissue specificity and regulation in response to pathogen attack or abiotic stress^23^. However, defensins from *Populus trichocarpa* have not yet been characterized.

Plants living in natural environments are vulnerable to various diseases due to their lack of mobility and a somatic immune system. Therefore, chemical fungicides are widely applied to improve the economic benefits of plant production. The use of chemical reagents improves plant biomass over a limited period of time, but also causes serious long-term problems. These problems can include increasing the risk of pathogen drug resistance, directly threatening the quality and safety of plant products, as well as polluting soil, rivers, lakes, and air to some extent. Therefore, improving plant resistance to pathogens as an alternative to chemical treatment has become a focus of research, with many promising directions for transgenic work. *AtPDF1*.*1* overexpression in *Arabidopsis thaliana* has been shown to result in decreased sensitivity to the non-host pathogen *Cercospora beticola*^24^. In addition, heterologous overexpression of a defensin from *Brassica juncea* was shown to significantly increase resistance to *Fusarium moniliforme* in *Nicotiana tabacum*^25^. Transferring the *DmAMP* gene from *Dahlia merckii* to *Oryza sativa* by transgenic technology promoted resistance to *Magnaporthe oryzae* and *Rhizoctonia solani*^26^. Overexpression of defensin from *Orychophragmus* in *Brassica napus* reduced damage to plants caused by *Sclerotinia sclerotiorum*^27^. The expression of *NmDef* from *Nicotiana megalosiphon* demonstrated that the *NmDef02* gene could improve resistance to *Phytophthora infestans* and *Alternaria solani* in *Solanum*^28^. A heterologous functional assay of defensin from *Solanum lycopersicum* showed that the transgenic *SlDef* gene significantly improved resistance to *Botrytis cinerea* in tomato^29^. However, it has not been confirmed whether the overexpression of defensin from *P*. *trichocarpa* (PtDef) promotes pathogen resistance in poplar species.

Salicylic acid (SA) and jasmonic acid (JA) play important roles in plant defense signal transduction, and their signaling pathways are closely related to plant resistance^30,31^. SA is an endogenous signal molecule that activates plant hypersensitive response (HR) and systemic resistance (SAR). SA can induce disease resistance, the expression of many disease-related proteins^32^, and abiotic stress resistance in plants. The response mechanism of SA to disease has been well studied in *Arabidopsis*. Non-expressor of pathogenesis-related gene 1 (NPR1) is the dominant regulator of acquired resistance in plants. Maintaining low SA concentrations in plant cells promotes NPR1 accumulation, thus activating the TGA transcription factors (WRKYs and Transcription factor TGAs) to activate the expression of plant resistance genes^33^. In the absence of SA, TGA2 and TGA5 can interact with NIMIN1, TOPLESS, the *CBNAC–SNI1* complex, and other components to inhibit PATHOGENESIS RELATED 1 (PR-1) promoter transcription^34^. NPR3 and NPR4 are also SA signal receptors; SA has higher affinity with NPR4 than with NPR3. When the SA concentration in plants is low, a combination of SA and NPR4 inhibits the degradation of NPR1 to maintain plant resistance. When plants are invaded by pathogens, different concentrations of SA accumulate around the infection site. A high SA concentration at the infection site binds NPR3, thus promoting NPR1 degradation, leading to plant cell death at the infection site and hindering the spread of pathogen infection^35^. JA, the key signal component of induced systemic resistance (ISR), accumulates rapidly and abundantly when plant tissues are invaded by pathogens. Studies have demonstrated that the coronatine insensitive 1/jasmonate ZIM-domain (COI1/JAZ) transcription factor signaling cascade pathway, an inhibitor of the JA pathway, is a key link in inducing plant pathogen resistance by JA and its derivatives. Jasmonoyl–L-isoleucine conjugates of JA and isoleucine form in plants under the influence of catalytic enzymes. JAZ proteins are thus degraded, relieving transcription factor inhibition and initiating JA response gene transcription^36^. SA and JA signal transduction mediation is known as the basic signal pathway for plant defense. Cross-talk between pathways induced by SA and JA and other signal transduction pathways can form a complex signal transduction network, allowing plants to respond quickly to different stimuli and speeding up defensive responses^37^.

In this study, we conducted molecular cloning, characterization, and functional analysis of the plant defensin PtDef from *P*. *trichocarpa*. Based on an analysis of its inhibition zone, we determined that the PtDef protein has antibacterial and antifungal activities. Defensins inhibited the growth and activity of *Agrobacterium tumefaciens* strain EHA105 to a greater extent when applied as an exogenous additive to rooting culture. Overexpression of *PtDef* resulted in field resistance to *Septotis populiperda* in transgenic poplars. Finally, we preliminarily explored the mechanism for enhanced disease resistance through *PtDef* gene overexpression, and determined that the SA and JA signal transduction pathways may be involved in the pathogen resistance response of poplar.

## Results

### *PtDef* molecular cloning and sequence analysis

Using *PtDef* sequence information (GenBank accession no. ABK93231.1), we designed specific primers and obtained the full length of *PtDef* (614 bp) by rapid amplification of cDNA ends. The *PtDef* gene had an ORF of 228 bp, encoding a *PtDef* polypeptide composed of 75 amino acids (Fig. S1A). The predicted Mw of the PtDef protein was 8.59 kD and its pI was 9.27. We found lower conservatism in the N-terminal than the C-terminal; both had eight conserved Cys structures (Fig. S1B), which contribute to the stability of the overall defensin spatial structure by allowing monomer proteins to form four disulfide bonds. This conservative Cys structure also exists in other species; their structural role appears to be universal and selected for in the evolutionary process. Indeed, the deduced PtDef amino acid sequence showed a high degree of homology with defensins from other plant species sequences, e.g., *Ziziphus jujuba* (XP_015879652.1, 78.67% identity) and *Vitis vinifera* (XP_002272913.2, 77.63% identity). A defensin phylogenetic tree was then constructed using the CLUSTAL W software and deduced amino acid sequences of PtDef and defensins from 19 other species. The defensin results classified 19 species into a single group, with a second group comprising only *Theobroma cacao* (GenBank accession no. EOY04853.1). The larger group was divided into four subgroups, suggesting that it may comprise multiple branches derived from an earlier ancestor (Fig. S1C). We then used the SWISS-MODEL server (http://www.expasy.org/swissmod/SWISS-MODEL.html) to construct the tertiary structures of the PtDef protein and *Arabidopsis* AtDEF proteins, and found that they were similar, consisting of three reverse-parallel β-pleated sheets and an α-helix (Fig. S1D,E).

### Prokaryotic expression, antibacterial activity, and antifungal activity assays

**Figure 1:**
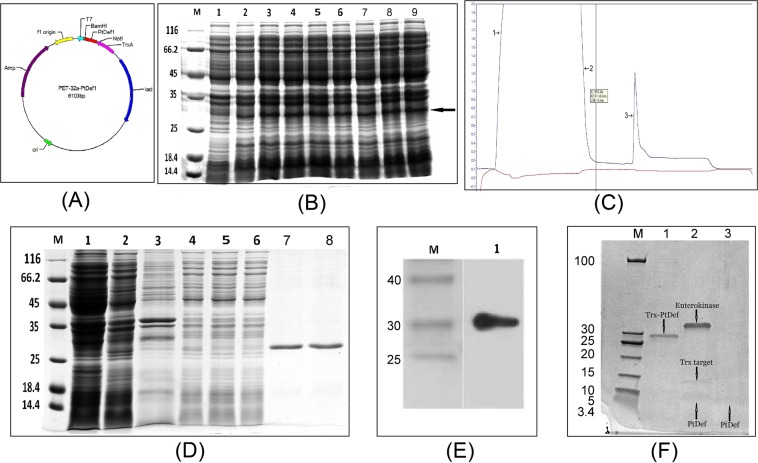
PtDef protein expression and purification. (**A**) Construction of the prokaryotic expression vector for PET-32a-*PtDef*. *BamH*I and *Not*I were used as restriction enzymes. (**B**) Analysis of the expressed fusion protein by 12% sodium dodecyl sulfate–polyacrylamide gel electrophoresis (SDS–PAGE). Lane M, molecular mass marker; lane 1, negative control; lanes 2–9, colonies 1–8; colonies were induced with 1 mM isopropyl-β-d-1-thiogalactopyranoside (the target protein was indicated by black arrow). The molecular weight (MW) of the target protein was about 27 kDa, and is indicated by the black arrow. (**C**) Ni-IDA affinity chromatography of the fusion protein using LP Data view (label 1, low-through; label 2, wash; label 3, elution.). (**D**) The analysis of purification of TrxA–PtDef fusion protein on a 12% SDS–PAGE gel. Lane M, MW marker; lane 1, negative control; lane 2, colonies were induced with 1 mM IPTG; lane 3, supernatant of cell lysate; lane 4, flow-through; lane 5, low-through; lane 6, wash; lane 7 and 8, elution. (**E**) Western blot analysis of purified TrxA–PtDef fusion protein using an mAb against the 6× His tag. Lane M, MW marker; lane 1, western blot. (**F**) Analysis of TrxA–PtDef fusion protein by 4–20% gradient SDS–PAGE. Analysis of TrxA–PtDef fusion protein following cleavage with enterokinase. Lane M, low-MW marker; lane 1, purified TrxA–PtDef fusion protein; lane 2, TrxA–PtDef cleavage products following TrxA digestion. Lane 3, purified recombinant PtDef.

Using our PET-32a plasmid and *PtDef* gene restriction enzyme site analysis results, the ORF of the *PtDef* gene was inserted into the prokaryotic PET-32a plasmid between the *Not*I and *BamH*I restriction sites (Fig. 1A). IPTG was added into the bacterial solution as an inducer, and 12% SDS–PAGE results revealed specific protein bands at about 27 kDa after induced expression, which was consistent with the theoretical calculation of protein size; however, no corresponding protein bands were detected at the corresponding location in the non-induced bacterial solution (Fig. 1B).

The target protein was detected in the supernatant and precipitation under induction conditions of 220 rpm and 37 °C for 4 h (Fig. 1D). The supernatant fusion protein was bound to Ni-IDA resin using 20 mmol/L imidazole, and the target protein was eluted using 250 mmol/L imidazole (Fig. 1C,D). To further explore whether the expressed product was a target protein, we performed Western blot analysis of the expressed product using mouse anti-6 × His antibody, with horseradish peroxidase-labeled sheep anti-mouse as the second antibody. The results showed that the expressed fusion protein was indeed the target protein (Fig. 1E).

Following successful expression, enterokinase was released from the fusion protein to release the target protein. Affinity chromatography was then used to isolate and purify the PtDef protein after enzymatic digestion and the affinity between Trx-6 × His and Ni^2+^ was used to remove the Trx-labeled protein through its binding with His TrapTMHP. The effluent of affinity chromatography contained the PtDef protein. Complete digestion was confirmed by 4–20% gradient gels (Fig. 1F).

**Figure 2:**
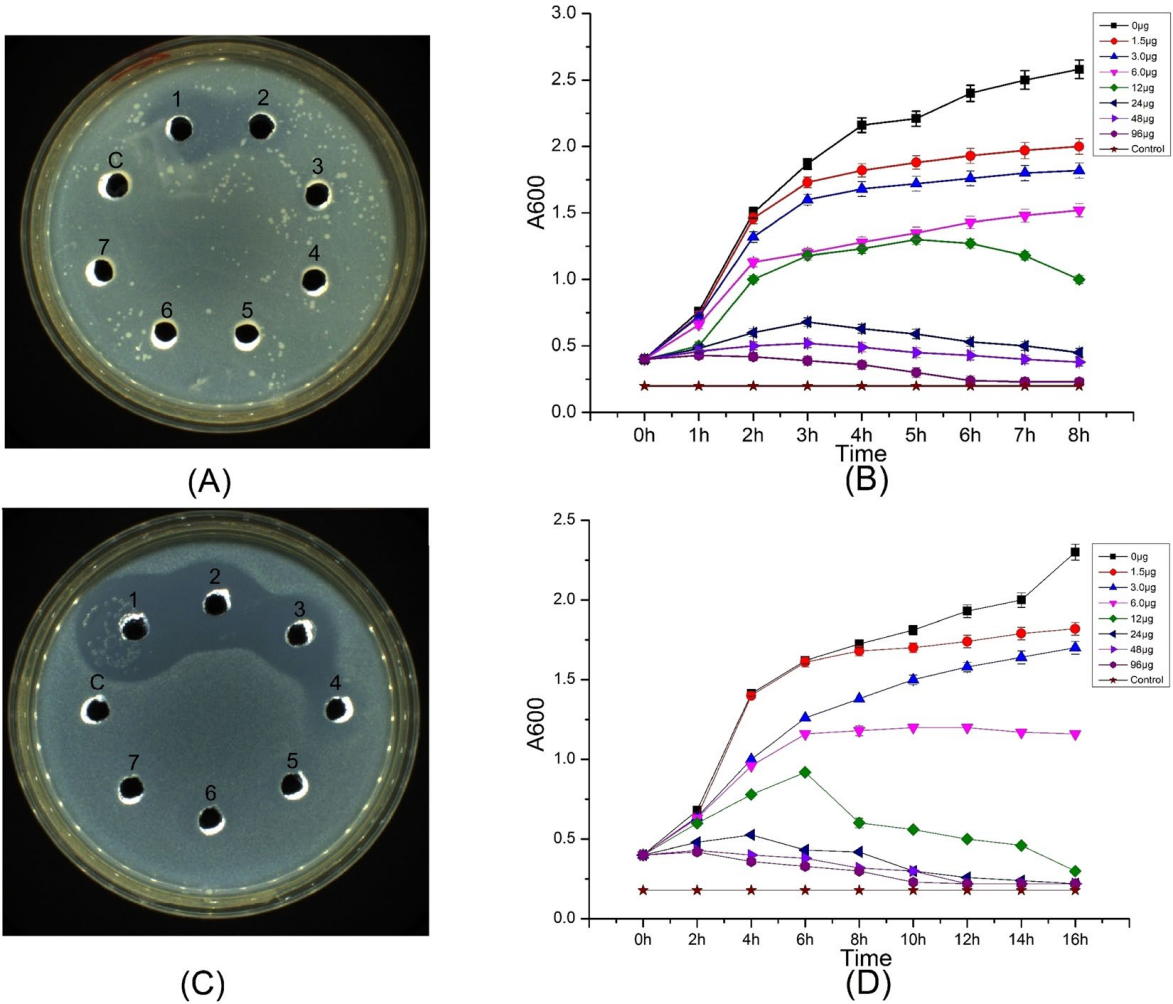
Analysis of antibacterial activity of purified PtDef protein and growth curves for *Escherichia coli* K12D31 and *Agrobacterium tumefaciens* EHA105 following the addition of different concentration of purified PtDef protein. (**A**) Anti-*E*. *coli* K12D31 activity of the purified PtDef protein. Lanes 1–7, PtDef protein levels at 1000, 500, 250, 125, 0.125, 62.5, and 31.25 ng/mL, respectively. C represents the 0.9% NaCl solution as a negative control. A 100-μL sample was loaded to each lane. (**B**) Eight groups of experiments were set up, and a gradient (0–96 μg) of PtDef protein treatments were added to the eight groups of *E*. *coli* K12D31 liquid medium. Bacterial concentration at 600 nm was measured at 1-h intervals for 8 h. Fresh medium (LB) was used as a control. Three experiments were carried out independently for each concentration. Values are the means ± standard deviation. (**C**) Anti-*A*. *tumefaciens* EHA105 activity of the purified PtDef protein. Lanes 1–7, TrxA–PtDef at 1000, 500, 250, 125, 0.125, 62.5, and 31.25 ng/mL, respectively. C represents the 0.9% NaCl solution as a negative control. A 100-μL sample was loaded to each lane. (**D**) Eight groups of experiments were set up and a gradient (0–96 μg) of PtDef protein treatments were added to the eight groups of *A*. *tumefaciens* EHA105 liquid medium. Bacterial concentration at 600 nm was measured at 2-h intervals for 16 h. Fresh medium was used as a control. Three experiments were carried out independently for each concentration. Values are the means ± standard deviation.

The minimum inhibitory concentration (MIC) and minimum bactericidal concentration were then determined and used to evaluate the antibacterial activity of the purified PtDef protein. The purified PtDef protein showed antimicrobial activity against *E*. *coli* K12D31 and *A*. *tumefaciens* EHA105; as the PtDef protein concentration increased, the number of bacteria decreased (Fig. 2A,C). These results showed that the MIC values of *E*. *coli* K12D31 and *A*. *tumefaciens* EHA105 differed, demonstrating that, although the *PtDef* protein was resistant to both bacteria, its sensitivity to each bacterial species differed, with much stronger activity against *A*. *tumefaciens* EHA105 than against *E*. *coli* K12D31. We then added 0–96 μg of purified PtDef protein to *E*. *coli* K12D31 cultures, and incubated the samples for 8 h. Compared with the control group, different concentrations of purified PtDef protein yielded different bacteriostatic growth effects; when ≥24 μg of purified PtDef protein was added, *E*. *coli* K12D31 growth was clearly inhibited (Fig. 2B). The same experiment was repeated with *A*. *tumefaciens* EHA105 for 16 h of incubation. Compared with the control group, bacterial growth was dose-dependent; *A*. *tumefaciens* EHA105 growth was clearly inhibited by exposure to ≥12 μg of purified PtDef protein (Fig. 2C). The inhibitory effect of the PtDef protein on *A*. *tumefaciens* EHA105 was greater than that on *E*. *coli* K12D31 (Fig. 2).

**Figure 3:**
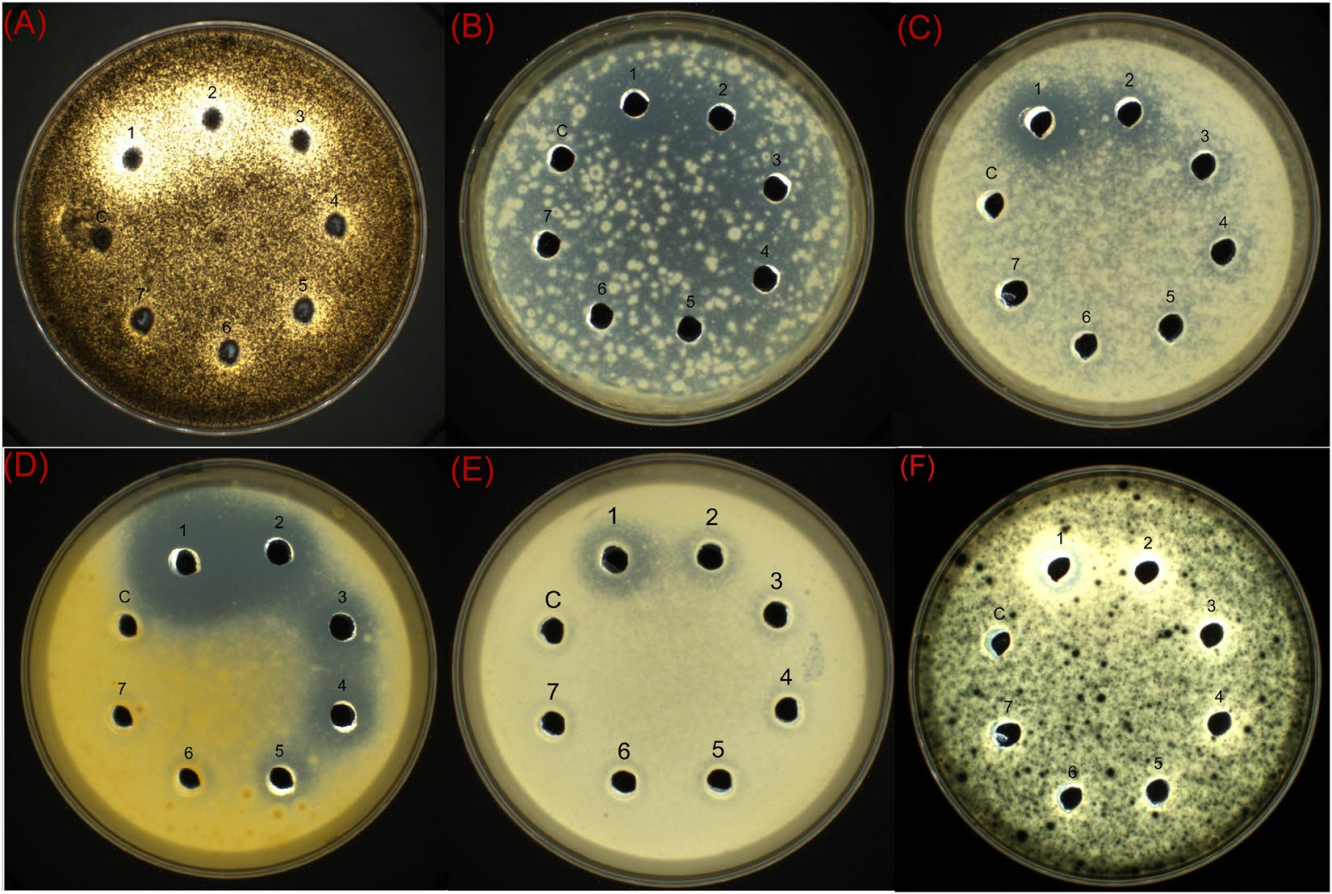
Bioactivity of purified PtDef protein against fungal species. Bioactivity of purified PtDef protein against (**A**) *Aspergillus niger*, (**B**) *Alternaria Nees*, (**C**) *Mucor corymbifer*, (**D**) *Marssonina populi*, (**E**) *Rhizopus* sp., and (**F**) *Neurospora crassa*. Lane C, 0.9% NaCl solution as a negative control. Lanes 1–7, purified PtDef protein (40, 20, 10, 5, 2.5, 1.25, and 0.625 μg/mL); a 100-μL sample was loaded in each lane.

To evaluate the antifungal activity of the purified PtDef protein, we applied treatments of 40, 20, 10, 5, 2.5, 1.25, and 0.625 μg/mL of protein in an agar well diffusion assay of six fungal species: *Aspergillus niger*, *Alternaria Nees*, *Mucor corymbifer*, *Marssonina populi*, *Rhizopus* sp., and *Neurospora crassa*. All treatments were performed in triplicate. We used the Scan 1200 (Interscience Laboratories Inc., Woburn, MA, USA) automatic colony counter to measure the diameters of bacteriostatic circles. Purified PtDef protein showed significant antifungal activity, with different inhibitory effects observed for different fungi at the same concentrations of purified PtDef protein. The greatest inhibitory effect was observed in *M*. *populi*, with a MIC diameter of 16 mm, whereas the MIC diameter of *Rhizopus* sp. was 10 mm (Figs 3 and S2).

### Evaluation of the purified PtDef protein as an analog of cefotaxime

We grew transgenic poplars in two rooting medium treatments, one containing 200 mg/L cefotaxime and the other containing 100 mg/L purified PtDef protein, and observed rooting and growth over a 30-day period. After 30 days, *A*. *tumefaciens* EHA105 was inhibited significantly when the purified PtDef protein as antibiotic analog, but the *A*. *tumefaciens* EHA105 was detected in MS medium containing the cefotaxime (Fig. S3). Thus, the purified PtDef protein acted as an effective antibiotic analog for cefotaxime to inhibit *A*. *tumefaciens* EHA105 growth.

**Figure 4:**
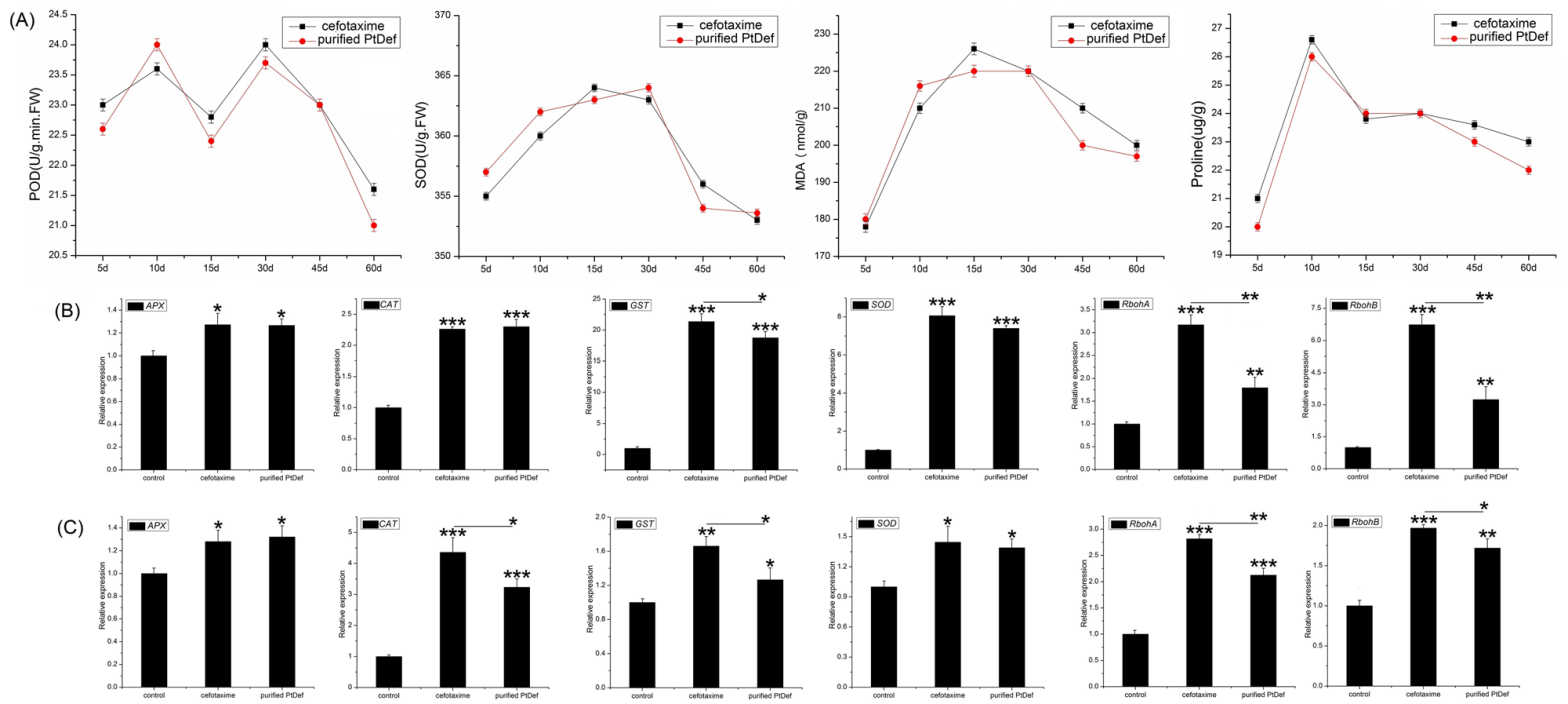
Evaluation of exogenous application of purified PtDef protein as an analog for cefotaxime in rooting culture medium. (**A**) Comparisons of the antioxidative activities of transgenic poplars grown in rooting medium with 100 mg/L purified PtDef protein and 200 mg/L cefotaxime. Peroxidase (POD), superoxide dismutase (SOD), malondialdehyde (MDA), and proline (Pro) activity of transgenic poplars grown in rooting medium with 200 mg/L of cefotaxime and 100 mg/L of purified PtDef protein. Three independent experiments were performed. All values are the means ± standard deviation. (**B**) Transcript levels of reactive oxygen species (ROS)-scavenging enzymes including ascorbate peroxidase (*APX*), catalase (*CAT*), glutathione S-transferase (*GST*), *SOD* and ROS-producing genes including *RbohA* and *RbohB* in leaves of transgenic poplars cultured in rooting culture medium with 100 mg/L of purified PtDef protein and 200 mg/L of cefotaxime, respectively. (**C**) The expression levels of ROS-scavenging molecules, including *APX*, *CAT*, *GST*, and *SOD*, and ROS-producing genes, including *RbohA* and *RbohB*, in roots of transgenic poplars cultured in rooting culture medium with 100 mg/L purified PtDef protein and 200 mg/L cefotaxime. Data were analyzed using quantitative reverse-transcription PCR and normalized to *actin* mRNA levels using the 2^−ΔΔCt^ method. Vertical bars represent the means ± standard deviation (n = 3). Significant differences are denoted by asterisks (*P < 0.05; **P < 0.01).

We compared MDA, Pro, POD, and SOD contents in leaves grown within each treatment; there were no significant differences in these components among treatments, suggesting that purified PtDef can be used as a substitute for cefotaxime in rooting culture medium to inhibit the growth of *A*. *tumefaciens* EHA105 in transgenic poplar (Fig. 4A).

Poplar leaves and roots were grown for 30 days in rooting medium (negative control), rooting medium with cefotaxime (positive control), and rooting medium with purified PtDef protein, and then removed. Genes encoding ROS-scavenging enzymes and those related to ROS generation were detected by qRT-PCR. The transcriptional levels of these genes increased significantly in the cefotaxime and purified PtDef treatments, indicating that these treatments simulated exogenous stress, which negatively affected poplar physiology (Fig. 4B,C). *GST*, *RbohA*, and *RbohB* expression were significantly lower in leaves from the purified PtDef treatment than in those from the cefotaxime treatment (Fig. 4B). *CAT*, *GST*, *RbohA*, and *RbohB* expression were significantly lower in roots from the purified PtDef treatment than in leaves from the cefotaxime treatment (Fig. 4C). We conclude that less toxicity is produced by the exogenous addition of purified PtDef than that of cefotaxime. These results demonstrate that the purified PtDef protein can be used as a biological antibiotic for plant tissue and tissue organ culture.

### Tissue-specific expression of *PtDef* in response to abiotic and biotic stress

**Figure 5:**
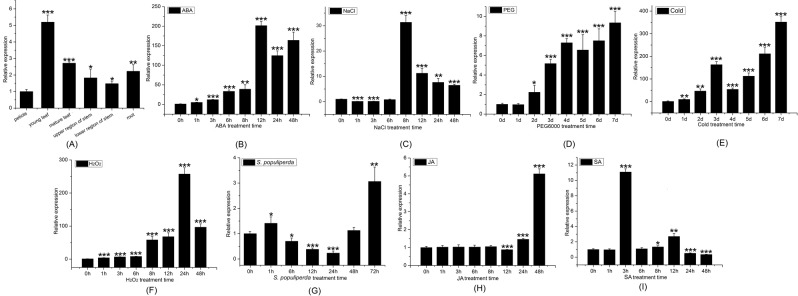
Analysis of *PtDef* gene expression patterns. (**A**) Expression analysis of the *PtDef* gene in various tissues, quantified by quantitative reverse-transcription (qRT)-PCR. Time series of *PtDef* expression in response to treatment with (**B**) 200 μM abscisic acid (ABA) during a period of 48 h, (**C**) 200 mM NaCl during a period of 48 h, (D) 10% PEG 6000 during a period of 48 h, (**E**) cold (4 °C) during a period of 7 days, (**F**) 2 mM H_2_O_2_ during a period of 48 h, and (**G**) *Septotis populiperda* during a period of 72 h. Data were analyzed by qRT-PCR and normalized to *actin* mRNA levels using the 2^−ΔΔCt^ method. Vertical bars represent the means ± standard deviation (n = 3). Significant differences are denoted by asterisks (*P < 0.05; **P < 0.01).

We used qRT-PCR to investigate *PtDef* expression in different tissues, including mature leaves, young leaves, petioles, roots, and the upper and lower regions of stems. *PtDef* expression was highest in leaves, and higher in young leaves than in mature leaves, probably because young leaves are rich in nutrients and susceptible to disease invasion. *PtDef* expression was also high in roots, perhaps because they are in direct contact with soil and easily invaded by soil diseases during plant growth (Fig. 5A). Thus, *PtDef* plays an important role in above- and below-ground poplar development.

With the extension of 200-μM ABA treatment duration, *PtDef* expression increased, reaching maximum expression at 12 h (Fig. 5B). When subjected to 200-mM NaCl treatment, *PtDef* expression decreased during the first 6 h, perhaps because the high NaCl concentration disrupted normal plant growth and required a minimum reaction time for its defense system to respond. However, after 8 h of NaCl treatment, *PtDef* expression reached a maximum (Fig. 5C). In the 10% PEG 6000 treatment, *PtDef* expression was significantly upregulated from days 2 to 7, with a peak appearing on day 7 (Fig. 5D). Under cold treatment, *PtDef* expression first increased, then decreased, and finally reached a maximum on day 7 (Fig. 5E). *PtDef* expression was upregulated during the first 48 h of 2-mM H_2_O_2_ treatment, reaching a maximum at 24 h (Fig. 5F). Under *Septotis populiperda* treatment, the highest expression level occurred at 72 h, followed by a decrease from 6 to 24 h (Fig. 5G), suggesting that *PtDef* expression was adjusted to alleviate plant damage following *S*. *populiperda* invasion. *PtDef* expression did not change significantly within the first 8 h of 200-μM JA treatment, but decreased significantly after 12 h, followed by a significant increase at 24 and 48 h (Fig. 5H). In the 200-μM SA treatment, *PtDef* expression reached a maximum (≥10 times higher than the control) at 3 h of induction (Fig. 5I). These results indicate that *PtDef* responds to a variety of biotic and abiotic stresses and can be used as a candidate gene for stress tolerance.

### Acquisition and identification of transgenic poplar plants

Transgenic poplar plants were screened and integration of the *PtDef* gene with poplar DNA was detected by PCR. Using 35S as a forward primer and the downstream of the *PtDef* gene as the reverse primer, a specific band was amplified in transgenic poplar, but not in the WT (Fig. S4A). We performed PCR using upstream and downstream primers of the *PtDef* gene, and produced a band >250 bp in the WT, perhaps due to introns in the poplar genome, whereas bands for the targeted gene appeared in the transgenic lines (Fig. S4B). We then performed qPCR to detect *PtDef* expression, and found that expression was significantly higher in the transgenic lines than in the WT, indicating that *PtDef* was successfully transcribed into the poplar genome and could transcribe intact mRNA (Fig. S4C).

### Growth differences between WT and transgenic poplars

**Figure 6:**
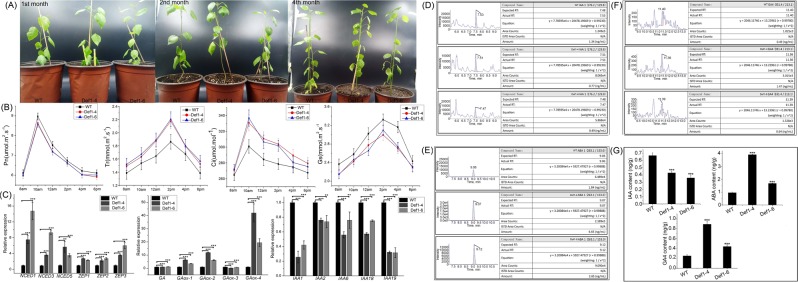
Phenotype and molecular level analysis of transgenic lines and wild-type (WT) poplars. (**A**) Phenotypes of 4-month-old transgenic and WT poplars. (**B**) Comparison of photosynthesis-related indicators, including net photosynthesis (*Pn*), intercellular CO_2_ concentration (*Ci*), transpiration (*Tr*), and stomatal conductance (*Gs*), between transgenic lines and WT poplars. (**C**) Transcript level analysis of abscisic acid (ABA)-, gibberellin (GA)-, and auxin (IAA)-related genes between transgenic lines and WT poplars. ABA-related genes included *NCED1*, *NCED3*, *NCED5*, *ZEP1*, *ZEP2*, and *ZEP3*. GA-related genes included *GA*, *GA20ox-1*, *GA20ox-2*, *GA20ox-3*, and *GA20ox-4*. IAA-related genes included *IAA1*, *IAA2*, *IAA6*, *IAA18*, and *IAA19*. Data were analyzed by quantitative reverse-transcription PCR and normalized to *actin* mRNA levels using the 2^−△△Ct^ method. Vertical bars represent the means ± standard deviation (n = 3). Significant differences are denoted by asterisks (*P < 0.05; **P < 0.01). High-performance liquid chromatography–tandem mass spectrometry was used to analyze differences between transgenic and WT poplars in (**D**) IAA content, (**E**) ABA content, and (**F**) GA4 content. (**G**) Statistical analyses of differences in IAA, ABA, and GA content between transgenic and WT poplars. Significant differences are denoted by asterisks (*P < 0.05; **P < 0.01).

Phenotypic analysis revealed no significant difference in growth, height, width, or leaf number between 4-month-old WT and transgenic poplars (Fig. 6A). qRT-PCR analysis showed lower expression of IAA-related genes, including *IAA1*, *IAA2*, *IAA6*, *IAA18*, and *IAA19*, in the transgenic lines Def1–1 and Def1–4 than in WT poplars (Fig. 6C). The expression of ABA-related genes, including 9-cis-epoxycarotenoid dioxygenase 1 (*NCED1*), *NCED3*, *NCED5* and zeaxanthin epoxidase 1 (*ZEP1*), *ZEP2*, and *ZEP3*, was significantly higher in transgenic lines that in WT poplars (Fig. 6C). Expression levels of GA-related genes were also higher in transgenic poplar lines (Fig. 6C).

The standard curves for IAA, GA, and ABA based on HPLC–MS/MS reference standard analysis are shown in Figs S5–7. IAA content was significantly lower (35–46%) in leaves from the transgenic lines than in those from WT poplars (Fig. 6D,G). *PtDef* gene overexpression in poplars significantly increased the ABA content of leaves (Fig. 6E,G). GA4 content was 2–3.5 times higher in transgenic lines than in WT poplars (Fig. 6F,G). Thus, although there was no difference in phenotype between transgenic and WT poplars within 4 months of growth, molecular and biochemical levels differed greatly between the poplar groups.

To further analyze differences in growth among poplar groups, we compared various photosynthesis parameters. *Gs* was lower and *Pn* slightly lower in transgenic lines than in WT poplars (Fig. 6B), whereas *Tr* and *Ci* were higher in transgenic lines than in WT poplars. Despite the phenotypic similarity among poplar groups, transgenic and WT poplars differed in molecular-level physiological indicators. Greater changes were observed in transgenic poplars than WT; thus, we expect phenotype to vary with the growth environment.

### JA-, SA-, LTP-, and PR-related gene expression analysis and comparison of JA and SA contents between WT and transgenic poplars

**Figure 7:**
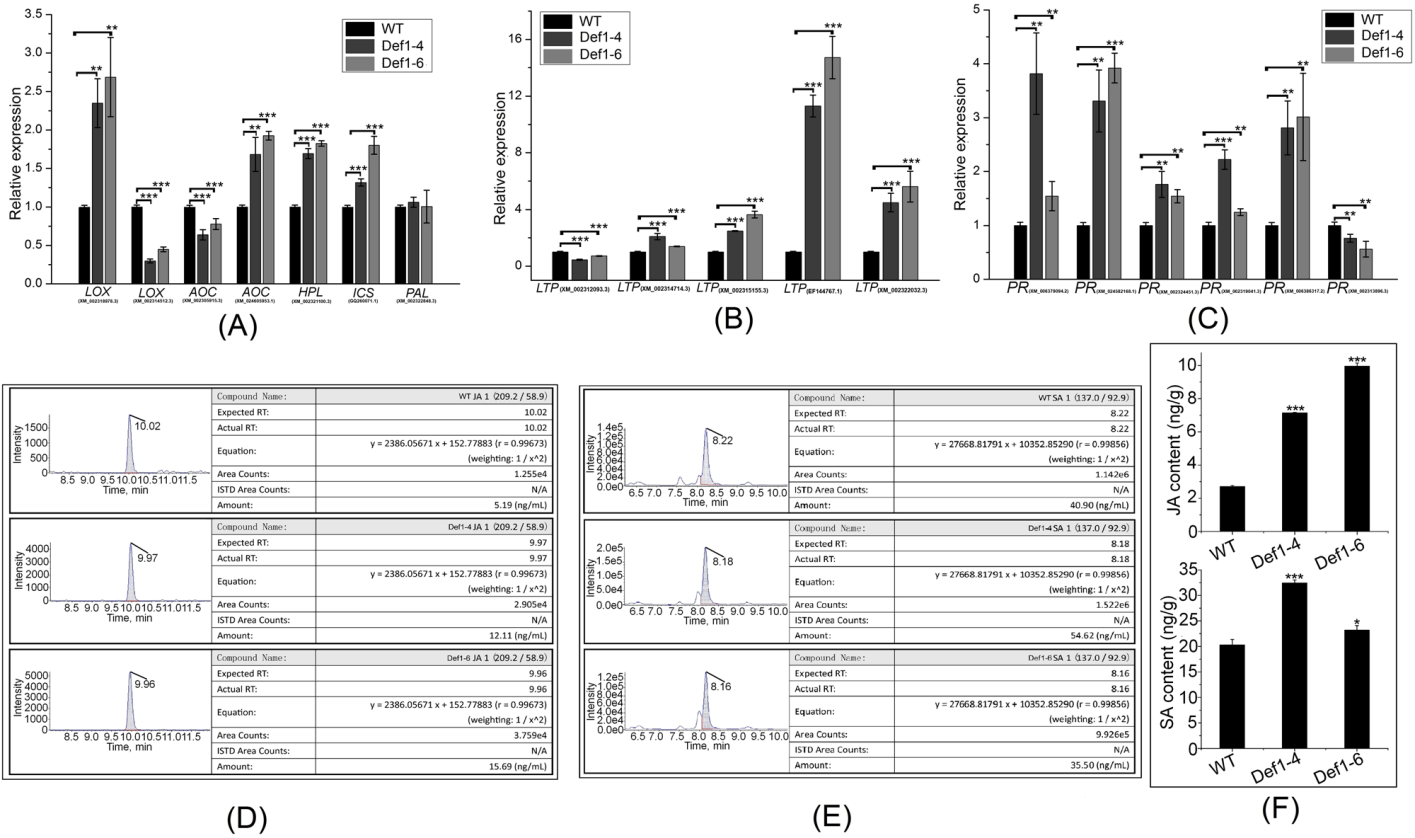
Transcript-level analysis of jasmonic acid (JA)-related, salicylic acid (SA)-related, pathogenesis-related (*PR*), and lipid-transfer protein (*LTP*) genes and JA and SA content between transgenic and wild-type (WT) poplars. (**A**) Transcript-level analysis of JA- and SA-related genes between transgenic and WT poplars. JA-related genes included *LOX*, *AOC*, and *HPL*. SA-related genes included *ICS* and *PAL*. (**B**) Transcript-level analysis of *PR* genes between transgenic and WT poplars. (**C**) Transcript-level analysis of *LTP* genes between transgenic and WT poplars. Data were analyzed by qRT-PCR and normalized to *actin* mRNA levels using the 2^−ΔΔCt^ method. Vertical bars represent the means ± standard deviation (n = 3). Significant differences are denoted by asterisks (*P < 0.05; **P < 0.01). Comparison of (**D**) JA and (**E**) SA content between transgenic and WT poplars using high-performance liquid chromatography–tandem mass spectrometry. (**F**) Statistical analysis of differences in JA and SA content between transgenic and WT poplars. Significant differences are denoted by asterisks (*P < 0.05; **P < 0.01).

Among the JA-related genes examined, the transcript levels of *LOC* XM_002318978.3, *AOC* XM_024605953.1, and *HPL* XM_002321100.3 were significantly higher in transgenic lines than in WT poplars. However, the expression levels of *LOC* XM_002314512.3 and *AOC* XM_002305915.3 were significantly lower in transgenic lines (Fig. 7A). Among the SA-related genes, *ICS* GQ260071.1 expression was significantly higher in transgenic lines, but there was no difference in *PAL* expression between poplar groups (Fig. 7A).

*PR* and *nsLTP* gene expression were compared between the transgenic and WT poplars using qRT-PCR analysis. The expression levels of all *nsLTP* genes were significantly higher in transgenic lines than in WT except *nsLTP* XM_002312093.3 (Fig. 7B). Expression levels of five of the six *PR* genes were higher in transgenic poplars, and that of one *PR* gene (XM_002313896.3) was lower (Fig. 7C).

The JA and SA standard curves prepared according to the HPLC–MS/MS results are shown in Figs S8 and S9. JA content was significantly lower in leaves of transgenic poplars than in WT leaves, which contained 1.7–2.7 times more JA (Fig. 7D,F). Overexpression of the *PtDef* gene significantly increased the SA content of leaves (Fig. 7D,F). These results suggest that *PtDef* overexpression affects the expression of JA- and SA-related genes, ultimately increasing JA and SA contents in poplar, which can subsequently affect plant disease resistance.

### *PtDef* overexpression enhances *S*. *populiperda* tolerance in transgenic poplars

**Figure 8:**
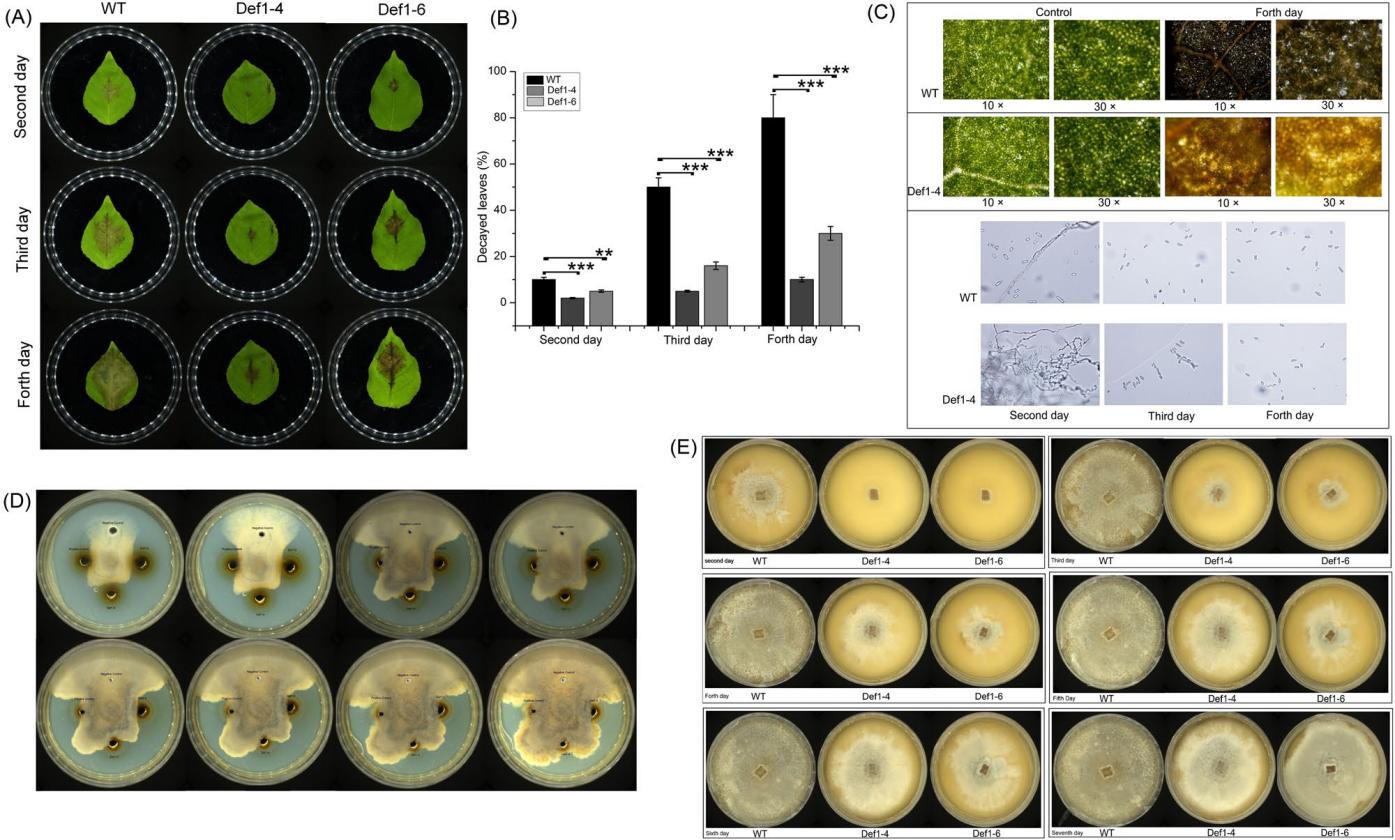
*Septotis populiperda* disease symptoms on poplars. (**A**) Time series and necrotic symptom analysis of transgenic and wild-type (WT) poplars. (**B**) Statistical analysis of the proportion of decayed leaf area to total leaf area following infection in transgenic and WT poplars. (**C**) Microscopic observation of changes in leaf internal structure and *S*. *populiperda* growth cycle following infection. (**D**) Analysis of total proteins in transgenic and WT poplars from days 2 to 9. (**E**) The effect of crude extraction on *S*. *populiperda* growth in transgenic and WT poplar leaves from days 2 to 7. Three independent experiments were performed.

Figure 8A shows the extent of *S*. *populiperda* spread on poplar leaves with time. Plaques were significantly smaller on leaves from transgenic lines (Def1–4 and Def1–6) than on those from WT plants on days 2, 3, and 4 (Fig. 8B), indicating that *PtDef* expression can improve poplar resistance to *S*. *populiperda*. We compared the degree of leaf damage in WT and Def1–4 transgenic poplar by microscopy. By day 4, many WT leaf cells had been destroyed, and the loss of integrity caused cellular fluid to flow out (Fig. 8C). However, in leaves from the Def1–4 line, cells remained closely arranged and intact, with no efflux of cellular fluid. We then examined *S*. *populiperda* morphology within a plaque by microscopy. By day 2, *S*. *populiperda* on WT leaves had produced conidia, whereas that on transgenic poplar remained in the mycelial state, producing conidia only on day 3 (Fig. 8C).

The *S*. *populiperda* inhibition zone was larger in leaves of the transgenic line than in those of WT (Fig. 8D), as *S*. *populiperda* grew more slowly near the pore where the *PtDef* gene was added. This result indicated that the transgenic protein inhibited *S*. *populiperda* growth better than WT endogenous proteins. Crude extracts of two transgenic lines (Def1–4 and Def1–6) and WT were added to PDA medium to cultivate and quantify *S*. *populiperda* growth for 7 days. We observed that *S*. *populiperda* extracted from WT grew faster, entirely covering the PDA medium by day 3. However, *S*. *populiperda* extracted from the transgenic lines grew slowly, covering the entire medium only at day 7 (Fig. 8E). These results demonstrate that *PtDef* overexpression in poplar significantly improves resistance to *S*. *populiperda*.

### Expression levels of genes related to JA and SA synthesis and PR genes following inoculation in transgenic and WT poplar

**Figure 9:**
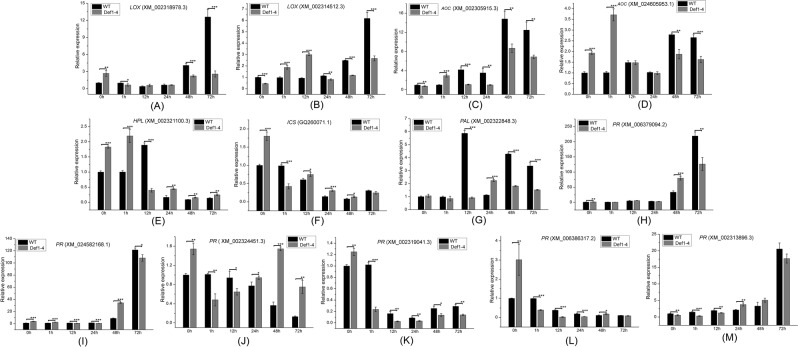
Comparative analysis of the expression of jasmonic acid (JA)- and salicylic acid (SA)-related, and pathogenesis-related (*PR*) genes in transgenic and wild-type (WT) poplars following inoculation with *Septotis populiperda*. Expression of (**A**) *LOX* (XM_002318978.3), (**B**) *LOX* (XM_002314512.3), (**C**) *AOC* (XM_002305915.3), (**D**) *AOC* (XM_024605953.1), (**E**) *HPL* (XM_002321100.3), (**F**) *ICS* (GQ260071.1), (**G**) *PAL* (XM_002322848.3), and (H–M) five *PR* genes in transgenic line Def1–4 and WT poplar following inoculation with *S*. *populiperda*. Data were analyzed by quantitative reverse-transcription PCR and normalized to *actin* mRNA levels using the 2^−ΔΔCt^ method. Vertical bars represent the means ± standard deviation (n = 3). Significant differences are denoted by asterisks (*P < 0.05; **P < 0.01).

The expression of *LOX* XM_002318978.3, a gene related to JA synthesis, first decreased and then increased following inoculation, and that of *LOX* XM_002318978.3 changed greatly in WT poplars; however, the expression of *LOX* XM_002318978.3 changed less in transgenic poplars (Fig. 9A). The expression of *LOX* XM_002314512.3 was upregulated 3 times in transgenic poplars, reaching a maximum at 12 h post-inoculation; in WT, its expression changed little post-inoculation, then increased to a peak at 72 h (Fig. 9B). The expression of *AOC* XM_002305915.3 was upregulated about 15 times in the WT, reaching a peak at 48 h after inoculation, but was upregulated only 9 times in transgenic poplars (Fig. 9C). *AOC* XM_024605953.1 expression reached a peak 1 h after inoculation in transgenic poplars, with a ratio of about 3.5, whereas it reached an expression peak 48 h after inoculation in WT, with a ratio of about 2.7 (Fig. 9D). The expression of *HPL* XM_002321100.3 reached a maximum 1 h after inoculation in transgenic poplars, and then decreased, whereas it reached a lower maximum 12 h after inoculation in WT poplar (Fig. 9E). These different expression levels of JA-related genes after inoculation in transgenic and WT poplars may indicate different JA content between poplar groups. JA forms a conjugate with isoleucine that can be catalyzed by an enzyme. When JAZ proteins are degraded, transcription factor inhibition is relieved and JA response gene transcription is initiated. This process may explain the improved disease resistance observed in transgenic lines.

Transcript levels of *ICS* and *PAL*, which are genes related to SA synthesis, were detected by qRT-PCR after inoculation. *ICS* expression in transgenic and WT poplars decreased after inoculation, reaching a minimum at 48 h (Fig. 9F). *PAL* expression reached a maximum 12 h after inoculation in WT poplar, with an upregulation rate of about 6. *PAL* expression in transgenic poplars reached a maximum at 24 h after inoculation, with an upregulation rate of about 2.5. The range of *PAL* expression was smaller in transgenic poplars than in WT (Fig. 9G). Maintaining low SA concentration in plant cells promotes NPR1 accumulation and activates TGA transcription factors (WRKYs and TGACG-SEQUENCE-SPECIFIC BINDING PROTEINs) to activate the expression of plant resistance genes^33^.

We then analyzed *PR* gene expression using qRT-PCR. The expression of *PR* XM_006379094.2 did not change significantly in either WT or transgenic poplars within 24 h after inoculation. At 48 h, *PR* XM_006379094.2 expression was 2.4 times higher in transgenic than in WT poplars; at 72 h, it reached a maximum in WT poplars, which differed significantly from that observed in transgenic poplars (Fig. 9H). *PR* XM_024582168.1 was upregulated after inoculation, with maximum expression observed in both transgenic and WT poplars at 72 h; these expression peaks were 120 and 108 times that of the control, respectively. However, *PR* XM_024582168.1 expression was 4 times higher in transgenic than in WT poplars at 48 h (Fig. 9I). *PR* XM_002324451.3 expression was downregulated within 72 h in WT poplars, but within 1–24 h in transgenic poplars, and returned to the original level at 48 h, and was again downregulated (Fig. 9J). The expression levels of *PR* XM_002319041.3 and *PR* XM_006386317.2 decreased within 72 h in both transgenic and WT poplars (Fig. 9K,L). *PR* XM_002313896.3 expression decreased and then increased in transgenic poplars, becoming 34% higher than that in WT poplars at 48 h, and then reaching a maximum at 72 h (Fig. 9M). These changes in the expression of *PR* genes and those related to SA synthesis after inoculation suggest that the SA signaling pathway may be involved in the poplar induction response to *Septotis populiperda*.

## Materials and Methods

### Bacterial strains, plants, fungi, vectors, adipose cells, and enzyme reagents

*E*. *coli* TOP10 strains were used for subcloning and *E*. *coli* BL21 (DE3) was used as the expression strain. A PET-32a plasmid containing *BamH*I and *Not*I restriction sites was purchased from Life Sensors (Malvern, PA, USA) for use as a prokaryotic expression vector, and PGWB9 (accession number AB289772) was used to construct the plant expression vector. Leaf disks from *Populus deltoides* × *Populus euramericana* ‘Nanlin895’ seedlings were prepared for transformation and precultured in 1/2 Murashige and Skoog (MS) medium. *A*. *tumefaciens* strain EHA105 was purchased from the Solarbio Bioengineering Institute (Beijing, China) and used to mediate poplar transformation. *Aspergillus niger*, *Alternaria Nees*, *Mucor corymbifer*, *Marssonina populi*, *Rhizopus* sp., and *Neurospora crassa* were obtained from the Institute of Microbiology at the Chinese Academy of Sciences. *S*. *populiperda* cells were kindly provided by Professors Zhu, respectively, at Nanjing Forestry University. *BamH*I and *Not*I restriction enzymes and T4 DNA ligase were purchased from Roche (Basel, Switzerland). A Gateway homologous recombination kit was purchased from Invitrogen (Carlsbad, CA, USA). All other reagents were purchased from the Nanjing Jiancheng Bioengineering Institute (Nanjing, China).

### RNA isolation, reverse-transcription (RT)-PCR and open reading frame (ORF) cloning

Total RNA was extracted using a plant-specific RNA kit (Invitrogen) according to the manufacturer’s instructions. First-strand cDNA was synthesized from RNA isolated from *P*. *trichocarpa* using M-MLV reverse transcriptase. Primers (Supplement Table 1) were designed and synthesized for defensin ORF cloning, the PCR system includes 2 µL forward and reverse primers, 2.0 µL cDNA as template, 5.0 µL 10× PCR buffer (Mg^2+^ plus), 1 µL 10 mM dNTPs, 0.5 µL rTaq DNA polymerase (Takara, Japan) and the ddH_2_O was to a constant volume up to 50 µL. Also, the PCR reaction was performed as follows: 95 °C for 10 min, 35 cycles of 95 °C for 30 s, 58 °C for 30 s, and 72 °C for 30 s and, finally, 72 °C for 10 min. Also the *PtDef* PCR product was purified (Takara, Japan) and inserted into the PEASY-T3 plasmid (TransGen Biotech, China). Subsequently, the positive clones were sequenced using the M13 primers. The nucleotide sequence, deduced amino acid sequence, and ORF of defensin from *P*. *trichocarpa* were analyzed using the ExPASy online package (http://www.expasy.org/translate/). We then calculated the molecular weight (Mw) of defensin and predicted its isoelectric point (pI) using the ExPASy pI/Mw tool (http://www.expasy.org/tools/pi_tool.html).

### Construction and expression of the recombinant plasmid

Primers with *BamH*I and *Not*I restriction sites were designed and synthesized (Supplement Table 1), the procedure of PCR was performed as below description and the *PtDef* product fused *BamH*I and *Not*I restriction sites obtain. Then, the *PtDef* PCR product and PET-32a plasmid were ligated together using the same sticky end after double digestion, and the recombinant plasmid mixture was transformed into *E*. *coli* TOP10 cells. Subsequently, PCR was used to identify the positive clones and the positive clones were sequenced using the T7 promoter primers.

The recombinant PET-32a-*PtDef* plasmid was transformed into *E*. *coli* BL21 (DE3). Positive colonies cultured in 1 L of lysogeny broth (LB) medium containing ampicillin were induced with 1.0 mM isopropyl-β-d-1-thiogalactopyranoside (IPTG) followed by incubation at 25 °C for 8 h. The uninduced solution, induced solution, supernatant, and sediment were analyzed using 12% sodium dodecyl sulfate–polyacrylamide gel electrophoresis (SDS–PAGE). Western blotting was performed according to the manufacturer’s instructions (Zhongshan Biotechnique, Beijing, China).

The fusion label thioredoxin (Trx) can help proteins fold correctly during the process of protein formation, to improve the expression of soluble proteins^38^. Therefore, thioredoxin fusion is among the most commonly used recombinant protein expression systems. TrxA–PtDef fusion protein purification was conducted using a nickel–nitrilotriacetic acid (Ni–NTA)-chelating column. Purified TrxA–PtDef fusion protein was incubated in the presence of an enterokinase (10 U) at 25 °C for 16 h. The solution was reapplied to the Ni column containing Ni–iminodiacetic acid (IDA) resin to remove His-tagged TrxA. The elution liquid and washing solution were analyzed using 4–20% gradient gels (Mini-R/PROTEAN TGX Gels; Bio-Rad, Hercules, CA, USA).

### Antibacterial activity and antifungal sensitivity assays

We performed an agar well diffusion assay to analyze the antibacterial activity of the purified PtDef protein. We analyzed the activity of the PtDef protein against *E*. *coli* K12D31 and *A*. *tumefaciens* EHA105 using the dilution method and configured seven gradient PtDef protein solutions. The microbial growth curve method was used to analyze the antibacterial activity of the PtDef protein, with the following steps: first, the cells were activated and cultured overnight; 80 μL fresh agrobacteria or bacteria solution was then added to 3 mL of LB growth medium on the next day. When the optical density at 600 nm (OD_600_) reached 0.6, we added 100 μL of purified PtDef solution at a concentration gradient (1.5–96 μg) to 3 mL LB growth medium (three replicates per group). Finally, the OD_600_ value was determined at the experimental point. We used 3 mL of LB growth medium without purified PtDef protein as a negative control (three replicates per group). We assessed antibacterial activity against *A*. *tumefaciens* EHA105 incubated at 28 °C and 220 rpm by determining the OD_600_ value after 2, 4, 6, 8, 10, 12, 14, and 16 h. We assessed the growth of *E*. *coli* K12D31 incubated at 37 °C and 220 rpm by measuring the OD_600_ after 1, 2, 3, 4, 5, 6, 7, and 8 h.

We analyzed antifungal activity by incubating six fungal species (*Aspergillus niger*, *Alternaria Nees*, *Mucor corymbifer*, *Marssonina populi*, *Rhizopus* sp., and *Neurospora crassa*) on potato dextrose agar (PDA) medium. We then dissolved 120 μL of purified PtDef protein in 0.9% NaCl (pH 7.0) to final concentrations of 40, 20, 10, 5, 2.5, 1.25, and 0.625 μg/mL and added these solutions to PDA medium plates (three replicates per experiment). As a negative control, we added 0.9% NaCl (pH 7.0) to the plates (three replicates per experiment). All plates were incubated at 23 °C for 72 h until mycelial growth was observed as pink, crescent-shaped samples. Agar diffusion was performed to determine antifungal activity^39^.

### Purified PtDef protein as an alternative to cefotaxime in the formation of transgenic poplars

Several methods have been used to inhibit the growth of *A*. *tumefaciens* EHA105 in transgenic operations; examples include cleaning leaves with MS liquid medium containing 200 mg/L cefotaxime and adding 200 mg/L cefotaxime during rooting medium screening. Generally, if the growth of *A*. *tumefaciens* EHA105 can be effectively suppressed during rooting medium screening, the success rate of transgenic procedures will be improved. The results of our defensin characterization research indicate that purified PtDef protein also substantially inhibits *A*. *tumefaciens* EHA105 growth. Therefore, we used defensin instead of cefotaxime for the transgenic processes in this study. Wild-type (WT) poplars (*Populus deltoides* × *Populus euramericana* ‘Nanlin895’) were selected for transgenic analysis using an efficient *Agrobacterium*-mediated transformation system^40^. In this experiment, 100 mg/L purified PtDef protein and 200 mg/L cefotaxime were added to the growth culture medium.

*A*. *tumefaciens* growth in rooting medium can lead to an increase in the production of reactive oxygen species (ROS), in turn causing oxidative damage to plant cells. Plant protective mechanisms include the production of secondary metabolites and enzymatic scavengers, including proline (Pro), superoxide dismutase (SOD), peroxidase (POD), malondialdehyde (MDA), ascorbate peroxidase (APX), catalase (CAT), and glutathione S-transferase (GST), which eliminate excessive ROS to maintain the reactive balance in plants cells. Therefore, the application of cefotaxime to rooting medium to inhibit *A*. *tumefaciens* growth also limits ROS accumulation. To investigate whether purified PtDef protein could similarly influence ROS accumulation, we established three treatment groups: rooting medium containing 100 mg/L purified PtDef protein as the experimental group, pure rooting medium as a negative control, and rooting medium containing 200 mg/L cefotaxime as a positive control. We compared the MDA, Pro, POD, and SOD contents of leaves grown within each treatment, and used a microplate reader (Bio-Rad) to detect activity levels of

### *PtDef* expression in various tissues in response to abiotic stress

To investigate *PtDef* transcription in different tissues, total RNA was isolated from mature leaves, young leaves, petioles, roots, and the upper and lower regions of stems of *P*. *trichocarpa*. *P*. *trichocarpa* seedlings treated with 200 mM NaCl, 200 μm abscisic acid (ABA), 200 μm SA, 200 μm JA, and 2 mM H_2_O_2_ were sampled at 0, 2, 4, 6, 8, 12, 24, and 48 h, and those subjected to 4 °C cold stress and 10% PEG 6000 were collected after 1, 2, 3, 4, 5, 6, and 7 days. In addition, a group of *P*. *trichocarpa* seedlings were wounded with *S*. *populiperda* and sampled at 0, 1, 3, 6, 9, 12, 24, 48, and 72 h. Following these treatments, leaves were immediately subjected to RNA extraction using a plant-specific RNA kit (Invitrogen). The housekeeping gene *actin* (XM_006370951.1) was used as an internal control for each reaction^41–44^. Triplicate measurements were used to determine the mean values for each parameter.

### Overexpression plasmid construction and transformation

Coding sequences of the *PtDef* gene were inserted into the pGWB9 vector using the homologous recombination method according to the manufacturer’s instructions (Invitrogen); the primers are listed in Supplement Table 1. We used the freeze–thaw method^45^ to introduce the recombinant plasmid pGWB9-*PtDef* into *A*. *tumefaciens* EHA105. Genetic transformation of the *PtDef* gene can be achieved by manipulation as previously described^40^; however, we modified this process for the present study, adding the PtDef protein as an exogenous additive to rooting culture medium to inhibit the growth of *A*. *tumefaciens* EHA105. We conducted RT-PCR and qRT-PCR analyses, and collected the putative transgenic lines to confirm that the *PtDef* gene had been successfully inserted into chromosomes and was stably expressed in cells. Regenerated shoots from the transgenic lines were recovered and transferred to half-strength MS rooting medium.

### Transplantation and growth comparison of transgenic and WT poplars

WT and transgenic poplars were obtained during the same growth period from subculture on half-strength MS medium and transferred to a mixture of equal quantities of soil, sterilized peat, and perlite (7:2:1) in a greenhouse. WT and transgenic poplar were cultured under long-day conditions (16-h light/8-h darkness). 23 °C and 74% humidity were kept constant during transplantation and plants were watered daily.

Plant hormones are small, simple molecular organic compounds with diverse and complex physiological effects. Plant hormones influence cell division, elongation, and differentiation to regulate plant germination, rooting, flowering, fruiting, sex determination, dormancy, and abscission, among other processes. To explore differences in the growth of WT and transgenic poplars, expression levels of auxin (IAA), gibberellin (GA), and ABA-related genes were measured using qRT-PCR analysis. To calculate standard curves for ABA, GA, and IAA standard solutions, we conducted high-performance liquid chromatography–tandem mass spectrometry (HPLC–MS/MS; Agilent 1290 HPLC system, Agilent Technologies, Santa Clara, CA, USA; QTRAP 6500 MS/MS system, SCIEX, Framingham, MA, USA). These curves were then used to compare the content of these phytohormones between transgenic and WT poplars. A concentration gradient of 0.1, 0.2, 0.5, 2, 5, 20, 50, and 200 ng/mL was used to prepare standard solutions of IAA, ABA, and GA with methanol (containing 0.1% formic acid). Points negatively affecting linearity were removed from the plot of the scalar equation. Liquid phase conditions were as follows: Poroshell 120 SB-C18 reversed-phase column (2.1 mm × 150 mm, 2.7 μm; Agilent); column temperature 30 °C; injection volume 2 L; mobile phase A:B = (methanol/0.1% formic acid):(water/0.1% formic acid); elution gradient 0–1 min: A = 20%, 1–9 min: A increased to 80%, 9–10 min: A = 80%, 10–10.1 min: A decreased to 20%, N, A = 20%). The mass spectrum conditions were as follows: curtain gas 15 psi, spray voltage 4500 V, atomizing gas pressure 65 psi, auxiliary gas pressure 70 psi, and atomizing temperature 204 °C.

To further explore quantitative differences between WT and transgenic poplars, we calculated photosynthesis-related indices, including net photosynthesis (*Pn*), intercellular CO_2_ concentration (*Ci*), transpiration (*Tr*), and stomatal conductance (*Gs*) using a portable photosynthetic apparatus (Li6400; LI-COR Biosciences, Lincoln, NE, USA).

### JA, SA, pathogenesis-related (PR), and lipid-transfer protein (LTP) gene expression and JA and SA content in transgenic and WT poplars

SA and JA are important endogenous signaling molecules that regulate plant disease resistance signaling pathways. Plants activate the expression of defense-related genes to improve resistance to biotic stress^46^. SA can induce plant resistance to pathogens and induce the expression of plant PR genes, further inducing SAR^47^. Non-specific LTPs (nsLTPs), which are also categorized as PR proteins, can inhibit the growth of pathogenic fungi and induce systemic resistance^48,49^. JA plays an important role as a plant hormone and signal compound in the processes of plant growth and development, particularly against biotic and abiotic stresses. JA can accumulate rapidly when plant tissues are attacked by pathogenic bacteria or insects; therefore, it is a key signaling molecule for ISR^50^.

Isochorismate synthase (*ICS*) and phenylalanine ammonia lyase (*PAL*) are important genes in the plant SA synthesis pathway; they are responsible for the conversion of branched acid and phenylalanine into isobranched acid and trans-cinnamic acid, respectively. Lipoxygenase (*LOX*), allene oxide cyclase (*AOC*), and fatty acid hydroperoxide lyase (*HPL*) are the corresponding genes for JA synthesis. Therefore, in this study, we determined JA and SA contents using an HPLC–MS/MS system. We also used qRT-PCR to detect transcript levels of the following *PR*s and *LTP*s: *PR XM_006379094*.*2*, *PR XM_024582168*.*1*, *PR XM_002324451*.*3*, *PR XM_002319041*.*3*, *PR XM_006386317*.*2*, *PR XM_002313896*.*3*, *LTP XM_002312093*.*3*, *LTP XM_002314714*.*3*, *LTP XM_002322032*.*3*, *LTP XM_002315155*.*3*, and *LTP EF144767*.*1*.

HPLC–MS/MS analysis was performed to create standard curves for the JA and SA contents in transgenic and WT poplars, using the same method and concentration gradient used to prepare the standard curves for phytohormones.

### Pathogen inoculation and disease response assay

*S*. *populiperda* was selected to analyze poplar disease response. Pathogens grown at 23 °C on PDA medium were activated for 1 week. Leaves from 4-month-old transgenic and WT poplars were punctured using a 5-mL syringe needle and inoculated with a small amount of PDA containing the pathogen or PDA as a negative control. Pathogenic soft rot symptoms were evaluated periodically. The maximal lesion size (diameter, mm) on leaves was recorded at 24–72 h as previously described^51^, and the degree of leaf damage was assessed in WT and transgenic poplars. The time of conidia appearance was determined in both groups by microscopy. Each experiment was repeated at least three times.

We performed *in vitro* assays of antifungal activity as described by Yuan *et al*.^52^. Leaves sampled from transgenic and WT poplars were used to extract total proteins according to the manufacturer’s instructions (Nanjing Jiancheng Bioengineering Institute). Total proteins were dissolved in 0.9% NaCl (pH 7.0) to a final concentration of 200 μg/mL and added to PDA medium plates (three replicates per experiment). We used 0.9% NaCl (pH 7.0) as a negative control (three replicates per experiment). All plates were incubated at 23 °C for 72 h until mycelial growth was observed. Extraction was performed on transgenic and WT poplar leaves using 70% acetone in liquid nitrogen. The crude extract was centrifuged and treated with ultrasound for 30 min; the residue was then re-extracted twice and the supernatant was mixed with chloroform. The same dose of crude extract was added to PDA medium, inoculated with *S*. *populiperda*, and cultured at 23 °C for 4 days. *S*. *populiperda* growth was then observed.

## Discussion

Like animals, plants have an intrinsic defense system. When plants are infected by external pathogens, their delicate immune defense systems are activated. This reaction is generally referred to as systemic induction of resistance in plants and confers broad-spectrum resistance to a range of unrelated pathogens. A large number of resistance compounds are synthesized in plants; antibacterial proteins including defensins are also induced to prevent further propagation and expansion of pathogenic bacteria^3,4,11,53,54^. Plant defensins are a class of cationic, alkaline, Cys-rich polypeptides with small Mw (about 5 kD, consisting of 45–54 amino acids) and a complex 3D structure. There are eight conservative Cys structures within the primary structure of plant defensins, forming four pairs of intrachain disulfide bonds. These four pairs of disulfide bonds are usually matched by Cys1–Cys8, Cys2–Cys5, Cys3–Cys6, and Cys4–Cys7. The other amino acid sequences are not conservative, but their advanced structures are very similar^55,56^. For example, the solution structure of a defensin (RsAFP1) isolated from *Raphanus sativus* was elucidated by nuclear magnetic resonance; it consists of three reverse-parallel β-sheets (Lys2 Arg6, His33 Tyr38, and His43 Pro50) and an α-helix (Asn18 Leu28), which form a very stable β–β structure with the help of four disulfide bonds^57^. The solution structures of defensins in *Pisum sativum* and *Vigna radiata* have also been elucidated. The basic structure of PsDef is an α-helix and three reverse-parallel β-sheets^58,59^. In this study, the predicted Mw of the PtDef protein (8.59 kD) was greater than that of the identified defensin protein. Through multiple sequence alignment, the N-terminal of defensins was relatively conservative aside from the eight conserved Cys structures. We also separately predicted the 3D structures of defensin proteins from *Arabidopsis* and *Populus trichocarpa*, and determined that the defensin consists of three reverse-parallel β-pleated sheets and an α-helix. These findings further demonstrate the conservativeness of defensins within their 3D structure.

Most plant defensins function to inhibit fungal growth, with half-maximal inhibitory concentration (IC_50_) values depending on the fungal species and the source of the plant defensin. For instance, the defensin from mustard (*WT1*) was expressed in *Nicotiana benthamiana*, and the protein purified by affinity chromatography. The IC_50_ of *WT1* against *Magnaporthe grisea* and *Botrytis cinerea* was 5 and 20 μg/mL, respectively. Meanwhile, the IC_50_ of *RsAFP2*, a defensin from radish seeds, against *M*. *grisea* was 0.1 μmol/L^9^. Besides their antifungal function, plant defensins also exhibit antibacterial activity. *WT1* showed no significant inhibitory effect on *Pseudomonas aeruginosa* at a concentration of 10 μg/mL; however, when the *WT1* concentration reached 20 μg/mL, it displayed a weak inhibitory effect on bacterial growth. In the current study, we first cloned the full-length genome of a defensin from *P*. *trichocarpa*, and then successfully obtained defensin proteins using a prokaryotic expression system and solubilizing tag (TrxA) on the PET-32a vector. Through bacteriostasis circle analysis, we found that PtDef inhibited the growth of two bacterial species and six tested fungi. PtDef inhibited the activity of *A*. *tumefaciens* EHA105 more strongly than that of *E*. *coli* K12D31. Antifungal activity also varied among target species; PtDef exhibited the highest inhibitory effect against *Marssonina populi*, and the lowest against *Rhizopus* sp. Antibiotics are widely used in transgenic processes, and their significant antibacterial effects can improve the efficiency of transgenic operations in plants. However, their use increases antibiotic resistance in some bacteria. In recent years, the search for suitable analogs to replace antibiotics has become a focus of research. Defensin is an ideal alternative to antibiotics due to its unique antibacterial mechanism and the inability of bacteria to develop resistance. In this study, PtDef strongly inhibited the growth of *A*. *tumefaciens* EHA105. To prevent the growth of agrobacteria, exogenous cefotaxime is typically applied to the rooting medium during the genetic modification process; therefore, we wondered whether PtDef could serve as an alternative to cefotaxime in the genetic modification process. We found that PtDef exhibited better anti-*Agrobacterium* activity than cefotaxime, and could reduce the need for medium replacement. Moreover, quantitative analysis showed that PtDef was less toxic than cefotaxime. Based on these results, the PtDef protein can be used instead of cefotaxime to inhibit *A*. *tumefaciens* EHA105 growth during the genetic modification process.

Previous studies have shown that defensins influence plant growth. For example, RsAFP2, MsDef2, and MsDef1 have been reported to have negative effects on plant growth^60,61^, whereas MtDef has been shown to have no effect on plant growth^62^. In the current study, when *PtDef* was overexpressed in poplar, there was no difference in phenotype between 4-month-old transgenic and WT poplars. However, molecular detection revealed significant differences between poplar groups. The expression of ABA- and GA-related genes was significantly higher in transgenic lines, but that of IAA-related genes was lower in transgenic lines. Plants lack the ability of active avoidance; however, they have evolved complex defense mechanisms in response to infection by disease. In recent years, research interest in the cloning, expression, and function of defensin has grown. Through in-depth studies of the mechanisms of plant-acquired resistance, researchers have found that defensins play an important role in plant resistance to pathogens. For example, overexpression of the defensin gene in transgenic *Oryza sativa* led to its improved resistance to *M*. *grisea*^63^. Overexpression of the defensin gene in transgenic potato, led to increased resistance to *B*. *cinerea*^64^. A defensin gene from *Medicago sativa* was transferred into a tomato genome, resulting in good resistance to *Fusarium wilt*^65^. The radish seed defensin gene *Rs2AFP2* was transferred into tobacco and tomato, significantly enhancing their resistance to *Alternaria longipes*^9^. Overexpression of the *alfAFP* peptide, isolated from seeds of *M*. *sativa*, in transgenic potato plants provided significant resistance to *Verticillium dahlia*^66^. Overexpression of *RsAFP2* in rice showed resistance to *Magnaporthe oryzae* and *Rhizoctonia solani*^26^. Overexpression of mustard defensins in tobacco also led to resistance to *Fusarium moniliform*^25^. However, transgenic plants incorporating defensin genes generally do not attain complete resistance to pathogenic fungi. To enhance resistance, other resistance genes can be transferred into plant cells in combination with the defensin, creating a synergistic effect^67^. Overexpression of two different AMPs from *Bombyx mori* in *Arabidopsis* enhanced its disease resistance^68^. An accumulation of antimicrobial genes in potato transgenic plants increased their resistance to bacterial and fungal pathogens^69^. In the current study, following inoculation with *S*. *populiperda*, the rate of disease occurrence was slower in transgenic lines than in WT poplars. For example, at 4 days post-inoculation, cells in leaves of the transgenic lines were able to maintain their integrity, whereas leaf cells of WT poplars were loosely aggregated and leaked cellular fluid. WT poplar leaves also developed conidia on day 2 post-inoculation, whereas the transgenic lines produced conidia one day later. Total protein and crude extracts from the transgenic lines also slowed the growth of *S*. *populiperda in vitro*.

JA and SA are the main hormones involved in plant resistance to disease invasion^31^. SA is an endogenous signaling molecule that activates plant HR and SAR, and induces the expression of many disease-related proteins^32^. Increased levels of endogenous SA enhance *PR* gene expression and plant resistance; exogenous SA application can stimulate the transcription of *PR* genes, increasing disease resistance^70^. We analyzed the expression of SA-related genes (*PAL* and *ICS*) and found that overexpression of the *PtDef* gene significantly increased that of the *ICS* gene. Based on the results of HPLC–MS/MS analysis, we found that SA content was significantly higher in *PtDef* transgenic poplar lines than in WT poplars. This result indicates that endogenous SA content increases in transgenic poplars, thereby inducing the expression of *PR* and *LTP* genes to enhance disease resistance in poplars. *LOX*, *AOC*, and *HPL* genes are related to JA synthesis; when plants are attacked by disease, genes that encode JA biosynthetase are upregulated^71^. In this study, the overexpression of defensin genes in poplar altered the expression of genes related to JA and SA synthesis. Two *LOX* genes and two *AOC* genes were detected in WT poplars and transgenic lines. One *LOX* gene (XM_002318978.3) and one *AOC* gene (XM_024605953.1) were up-regulated in transgenic lines, but those of another *LOX* gene (XM_002314512.3) and another *AOC* gene (XM_002305915.3) were downregulated. The expression of *HPL* gene XM_002321100.3 was significantly higher in transgenic lines.

HPLC–MS/MS also showed that overexpression of the *PtDef* gene improved JA content in poplars. JA is a key endogenous growth regulator of the plant defense response to pathogenic microorganisms, and plays an important role in signal transduction of the system defense response to increase plant disease resistance. We observed a synergistic reaction^72^ between the SA signaling pathway and the JA signaling pathway in poplar; both of these pathways can induce resistance to pathogens, and the combination of SAR and ISR improves the plant’s ability to control disease^73^. Hormone signaling molecules can form a complex defense system in the face of pathogen infection, including the distribution and effective regulation of components, to enhance the efficiency of the plant defense response. We also examined changes in the expression of *PR* genes and SA- and JA-related genes after inoculation with pathogens, and observed significant differences in expression levels between transgenic and WT poplars. This result indicates that the SA and JA signal transduction pathway in poplar displays synergistic and antagonistic effects similar to those in *Arabidopsis*^72,74^. Our study of the SA and JA signal transduction pathways in poplars represents a preliminary exploration; further study is required to elucidate their interaction.

## Conclusion

In conclusion, we cloned a full-length gene encoding *PtDef*, and investigated its expression patterns in different tissues of *P*. *trichocarpa* and under different stresses. We also purified the PtDef protein based on prokaryotic expression and purification technology; purified PtDef inhibited the growth of *E*. *coli* K12D31, *A*. *tumefaciens* EHA105, and six tested fungal species. Purified PtDef was a suitable alternative to cefotaxime as an exogenous additive to rooting culture medium, as shown by its inhibition of *A*. *tumefaciens* EHA105 production in rooting culture medium. Such an application would improve the efficiency of obtaining transgenic plants. Overexpression of *PtDef* significantly increased poplar resistance to *S*. *populiperda* based on phenotype analysis and the expression of *PR* and *LTP* genes, as well as JA- and SA-related genes. Finally, we performed a preliminary exploration of the SA and JA signaling pathways for enhanced resistance in transgenic poplars, and observed synergistic or antagonistic effects, which together regulate disease resistance in poplars.

## Supplementary information

Supplementary 1

Supplementary 2

Supplementary 3

Supplementary 4

Supplementary 5

Supplementary 6

Supplementary 7

Supplementary 8

Supplementary 9

Supplementary 10

Supplementary table
