# Supplementary material for: Characterization, expression profiling, and functional analysis of a Populus trichocarpa defensin gene and its potential as an anti-Agrobacterium rooting medium additive
Source: Sci Rep. 2019 Oct 25;9:15359. doi: 10.1038/s41598-019-51762-0 (PMC6814764; doi:10.1038/s41598-019-51762-0)
Supplement: Supplementary file 1 — Supplementary 1 [file 41598_2019_51762_MOESM1_ESM.pdf]

Characterization, expression profiling, and functional analysis of a *Populus trichocarpa* defensin gene and its potential as an anti-*Agrobacterium* rooting medium additive

Hui Wei[1,a], Ali Movahedi[1,a], Chen Xu[1,2,a], Weibo Sun[1,a], Lingling Li[1], Dawei Li[1], Qiang Zhuge[1]*

[1]Co-Innovation Center for Sustainable Forestry in Southern China, Key Laboratory of Forest Genetics & Biotechnology, Ministry of Education, College of Biology and the Environment, Nanjing Forestry University. Nanjing 210037, China

[2]Jiangsu Provincial Key Construction Laboratory of Special Biomass Resource Utilization, Nanjing Xiaozhuang University, Nanjing, 211171, China

*Correspondence should be addressed to Qiang Zhuge: Co-Innovation Center for Sustainable Forestry in Southern China, Key Laboratory of Forest Genetics and Biotechnology, Ministry of Education, College of Biology and the Environment, Nanjing Forestry University, Nanjing 210037, China. E-mail: qzhuge@njfu.edu.cn; Fax: +86 25 85428701

[a] These authors are contributed equally as the first author

Hui Wei: 15850682752@163.com
Ali Movahedi: ali_movahedi@njfu.edu.cn
Chen Xu: xuchenidea@hotmail.com
Weibo Sun: cz851115@126.com
Lingling Li: 1162520689@qq.com
Dawei Li: dwli@njfu.edu.cn

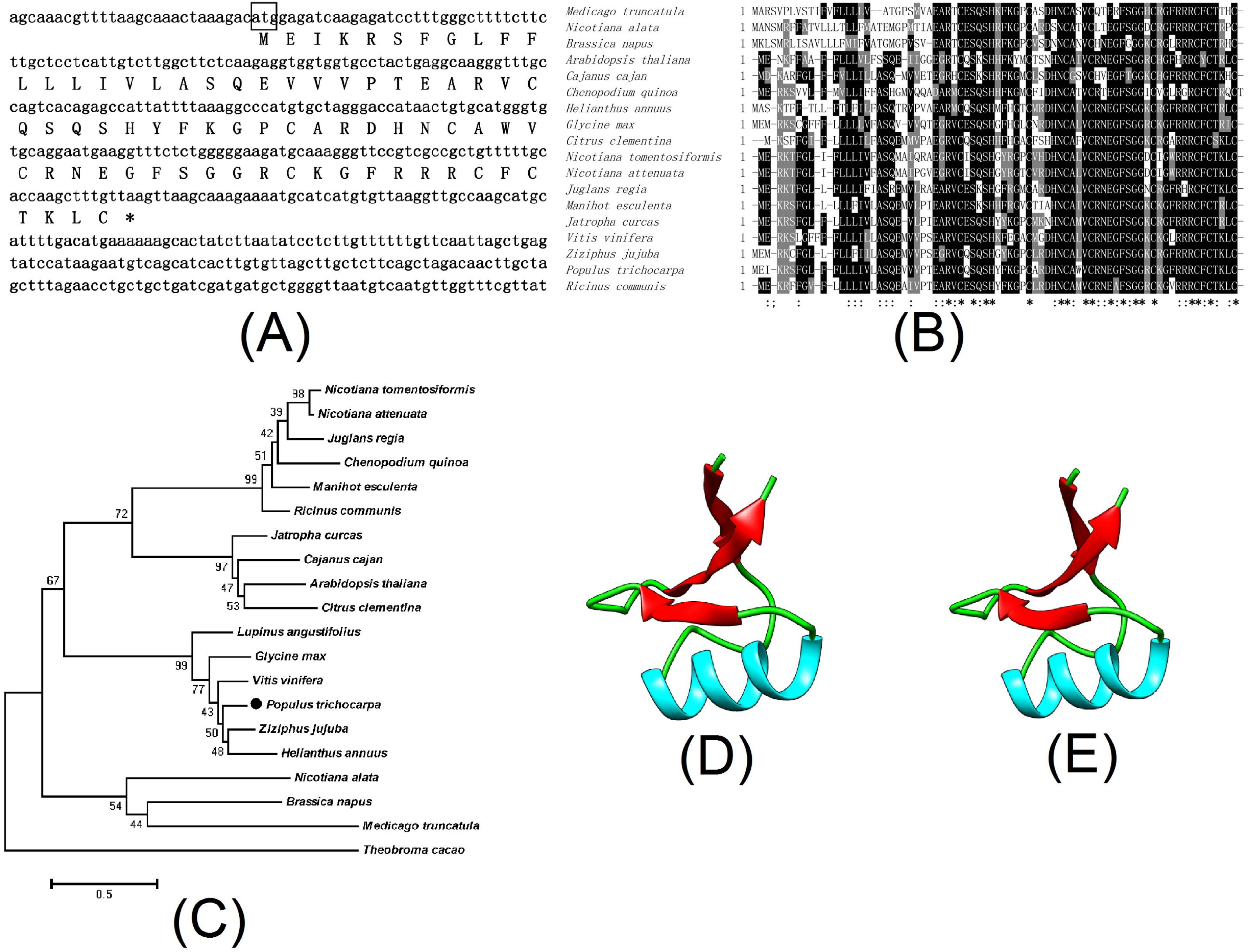
agcaaacgttttaagcaaactaaagacatggagatcaagagatcctttgggcttttcttc
M E I K R S F G L F F
ttgctcctcattgtcttggcttctcaagaggtggtggtgcctactgaggcaagggtttgc
L L L I V L A S Q E V V V P T E A R V C
cagtcacagagccattattttaaaggcccatgtgctagggaccataactgtgcatgggtg
Q S Q S H Y F K G P C A R D H N C A W V
tgcaggaatgaaggtttctctgggggaagatgcaaagggttccgtcgccgctgtttttgc
C R N E G F S G G R C K G F R R R C F C
accaagctttgttaagttaagcaaagaaaatgcatcatgtgttaaggttgccaagcatgc
T K L C *
atttgacatgaaaaaagcactatcttaatatcctcttgtttttgttcaattagctgag
tatccataagaatgtcagcatcacttgtgttagcttgctcttcagctagacaacttgcta
gctttagaacctgctgctgatcgatgatgctggggttaatgtcaatgttggtttcgttat
(A)
Medicago truncatula
Nicotiana alata
Brassica napus
Arabidopsis thaliana
Cajanus cajan
Chenopodium quinoa
Helianthus annuus
Glycine max
Citrus clementina
Nicotiana tomentosiformis
Nicotiana attenuata
Juglans regia
Manihot esculenta
Jatropha curcas
Vitis vinifera
Ziziphus jujuba
Populus trichocarpa
Ricinus communis
(B)
Nicotiana tomentosiformis
Nicotiana attenuata
Juglans regia
Chenopodium quinoa
Manihot esculenta
Ricinus communis
Jatropha curcas
Cajanus cajan
Arabidopsis thaliana
Citrus clementina
Lupinus angustifolius
Glycine max
Vitis vinifera
Populus trichocarpa
Ziziphus jujuba
Helianthus annuus
Nicotiana alata
Brassica napus
Medicago truncatula
Theobroma cacao
0.5
(C)
(D)
(E)

Figure S1. Characterization of the PtDef gene. (A) Nucleotide and deduced amino acid sequences of PtDef (GenBank accession no. ABK93231.1). The complete deduced amino acid sequence is depicted in single-letter code beneath the corresponding nucleotide sequence. The initiation codon is boxed and the termination codon is indicated by an asterisk (*). (B) Comparison of the deduced amino acid sequences of the conserved regions of Populus trichocarpa, PtDef, and corresponding parts of other known defensins. Asterisks (*) indicate that the aligned residues are identical. Conserved or semi-conserved substitutions, as determined by the ClustalWare software, are marked by (:) and (.), respectively. (C) Phylogenetic tree showing the relationships between the P. trichocarpa defensin PtDef (ABK93231.1) amino acid sequence and other identified Def sequences. The tree was constructed using the neighbor-joining (NJ) method with the MEGA 5.1 software and bootstrapped 1,000 times. Bootstrap percentages are indicated at the branch points. In all cases, tree topologies obtained using the NJ, minimum evolution, and maximum parsimony methods were identical. The accession numbers of Def sequences obtained from GenBank were as follows: Arabidopsis thaliana (NP_201171.1), Brassica napus (AGT51229.1), Cajanus cajan (XP_020213715.1), Chenopodium quinoa (XP_021733386.1), Citrus clementina (XP_006426420.1), Glycine max (XP_001336660.1), Helianthus annuus (XP_022010890.1), Jatropha curcas (XP_012079607.1), Juglans regia (XP_018816007.1), Lupinus angustifolius (XP_019414592.1), Manihot esculenta (XP_021619048.1), Medicago truncatula (XP_003628977.1), Nicotiana alata (AOD75394.1), Nicotiana attenuata (XP_019259802.1), Nicotiana tomentosiformis (XP_009590912.1), Ricinus communis (EEF49129.1), Theobroma cacao (EOY04853.1), Vitis vinifera (XP_002272913.2), and Ziziphus jujuba (XP_015879652.1). (D) Tertiary structure prediction of defensin from Arabidopsis (NP_201171.1). In the drawing, α-helices, β-strands, and random coils are highlighted in cyan, red, and green, respectively. (E) Tertiary structure prediction of defensin from P. trichocarpa (ABK93231.1). In the drawing, α-helices, β-strands, and random coils are highlighted in cyan, red, and green, respectively.
