# Supplementary material for: Characterization, expression profiling, and functional analysis of a Populus trichocarpa defensin gene and its potential as an anti-Agrobacterium rooting medium additive
Source: Sci Rep. 2019 Oct 25;9:15359. doi: 10.1038/s41598-019-51762-0 (PMC6814764; doi:10.1038/s41598-019-51762-0)
Supplement: Supplementary file 2 — Supplementary 2 [file 41598_2019_51762_MOESM2_ESM.pdf]

Characterization, expression profiling, and functional analysis of a *Populus trichocarpa* defensin gene and its potential as an anti-*Agrobacterium* rooting medium additive

Hui Wei[1,a], Ali Movahedi[1,a], Chen Xu[1,2,a], Weibo Sun[1,a], Lingling Li[1], Dawei Li[1], Qiang Zhuge[1]*

[1]Co-Innovation Center for Sustainable Forestry in Southern China, Key Laboratory of Forest Genetics & Biotechnology, Ministry of Education, College of Biology and the Environment, Nanjing Forestry University. Nanjing 210037, China

[2]Jiangsu Provincial Key Construction Laboratory of Special Biomass Resource Utilization, Nanjing Xiaozhuang University, Nanjing, 211171, China

*Correspondence should be addressed to Qiang Zhuge: Co-Innovation Center for Sustainable Forestry in Southern China, Key Laboratory of Forest Genetics and Biotechnology, Ministry of Education, College of Biology and the Environment, Nanjing Forestry University, Nanjing 210037, China. E-mail: qzhuge@njfu.edu.cn; Fax: +86 25 85428701

[a] These authors are contributed equally as the first author

Hui Wei: 15850682752@163.com
Ali Movahedi: ali_movahedi@njfu.edu.cn
Chen Xu: xuchenidea@hotmail.com
Weibo Sun: cz851115@126.com
Lingling Li: 1162520689@qq.com
Dawei Li: dwli@njfu.edu.cn

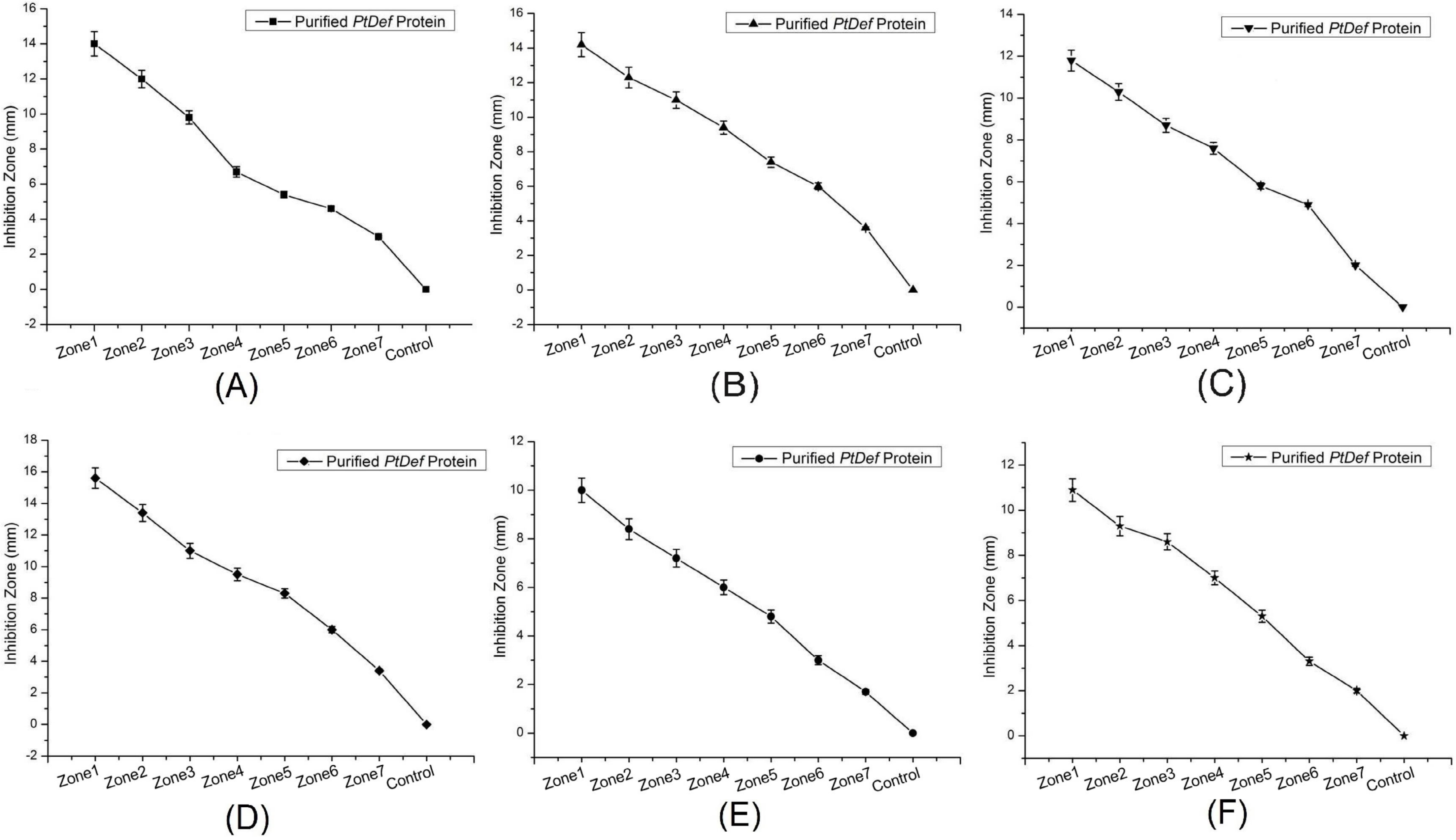
Purified PtDef Protein
Inhibition Zone (mm)
Zone1
Zone2
Zone3
Zone4
Zone5
Zone6
Zone7
Control
(A)
(B)
(C)
(D)
(E)
(F)

Figure S2. Treatment with different concentrations of the purified PtDef protein yielded bacterial inhibition zones of different sizes for (A) Aspergillus niger, (B) Alternaria Nees, (C) Mucor corymbifer, (D) Marssonina populi, (E) Rhizopus sp., (F) Neurospora crassa. Lane C, 0.9% NaCl solution as a negative control. Lanes 1–7, purified PtDef protein treatment at 40, 20, 10, 5, 2.5, 1.25, and 0.625 μg/mL, respectively; an 80-μL sample was loaded in each lane. Three independent experiments were performed.
