# Supplementary material for: Characterization, expression profiling, and functional analysis of a Populus trichocarpa defensin gene and its potential as an anti-Agrobacterium rooting medium additive
Source: Sci Rep. 2019 Oct 25;9:15359. doi: 10.1038/s41598-019-51762-0 (PMC6814764; doi:10.1038/s41598-019-51762-0)
Supplement: Supplementary file 4 — Supplementary 4 [file 41598_2019_51762_MOESM4_ESM.pdf]

Characterization, expression profiling, and functional analysis of a *Populus trichocarpa* defensin gene and its potential as an anti-*Agrobacterium* rooting medium additive

Hui Wei[1,a], Ali Movahedi[1,a], Chen Xu[1,2,a], Weibo Sun[1,a], Lingling Li[1], Dawei Li[1], Qiang Zhuge[1]*

[1]Co-Innovation Center for Sustainable Forestry in Southern China, Key Laboratory of Forest Genetics & Biotechnology, Ministry of Education, College of Biology and the Environment, Nanjing Forestry University. Nanjing 210037, China

[2]Jiangsu Provincial Key Construction Laboratory of Special Biomass Resource Utilization, Nanjing Xiaozhuang University, Nanjing, 211171, China

*Correspondence should be addressed to Qiang Zhuge: Co-Innovation Center for Sustainable Forestry in Southern China, Key Laboratory of Forest Genetics and Biotechnology, Ministry of Education, College of Biology and the Environment, Nanjing Forestry University, Nanjing 210037, China. E-mail: qzhuge@njfu.edu.cn; Fax: +86 25 85428701

[a] These authors are contributed equally as the first author

Hui Wei: 15850682752@163.com
Ali Movahedi: ali_movahedi@njfu.edu.cn
Chen Xu: xuchenidea@hotmail.com
Weibo Sun: cz851115@126.com
Lingling Li: 1162520689@qq.com
Dawei Li: dwli@njfu.edu.cn

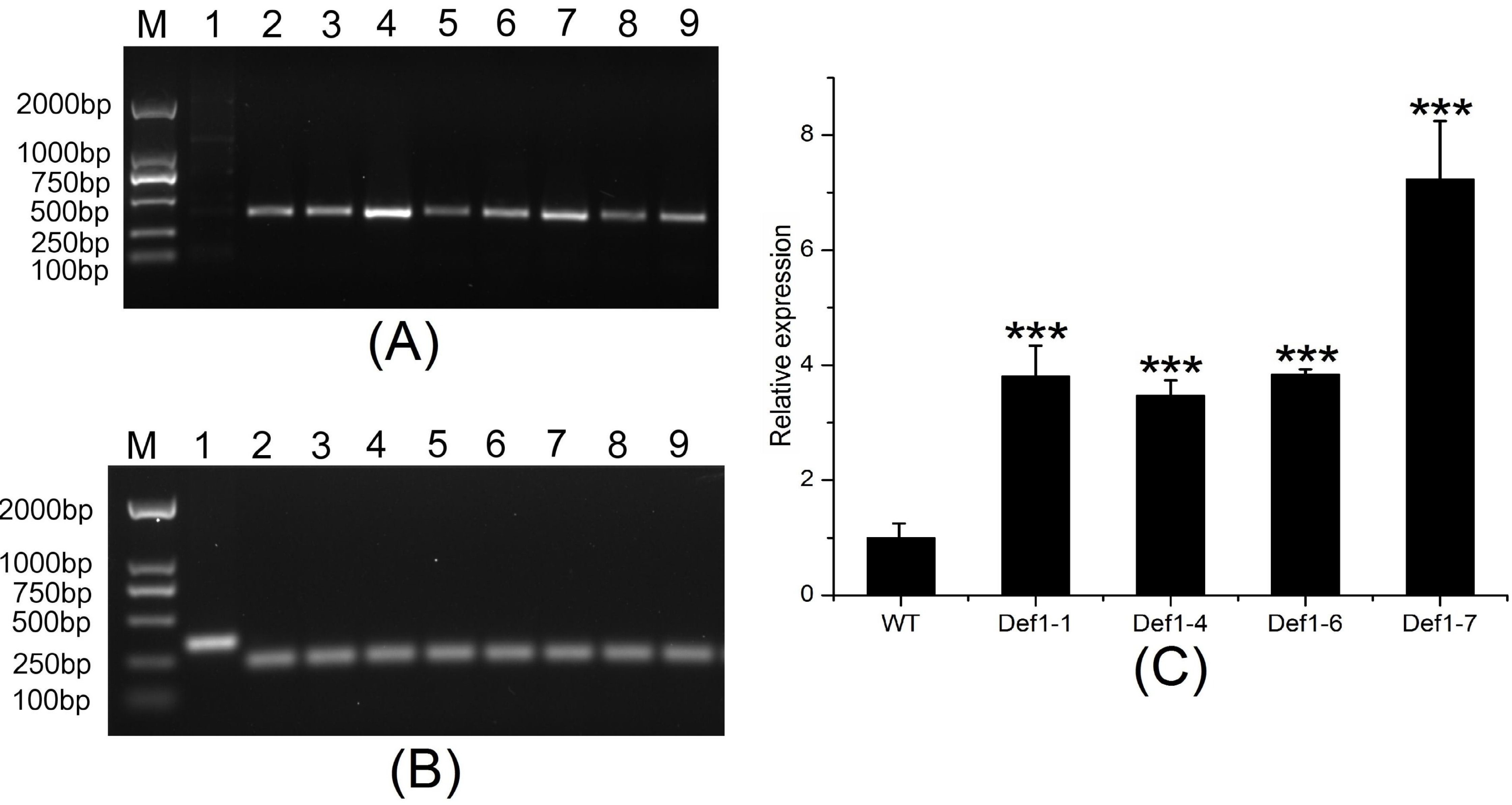

M 1 2 3 4 5 6 7 8 9
2000bp
1000bp
750bp
500bp
250bp
100bp
(A)
M 1 2 3 4 5 6 7 8 9
2000bp
1000bp
750bp
500bp
250bp
100bp
(B)
Relative expression
0
2
4
6
8
***
WT
Def1-1
Def1-4
Def1-6
Def1-7
(C)

Figure S4. Molecular identification of poplar plants exhibiting PtDef overexpression. (A) Detection of the PtDef gene in transgenic and wild-type (WT) poplars by PCR using the genome as a template, with the 35S promoter as the upstream primer and PtDef-R as the downstream primer. (B) Detection of the PtDef gene in transgenic and WT poplars by PCR using the genome as a template, with PtDef-F and PtDef-R the upstream and downstream primers, respectively. (C) Detection of PtDef gene expression in transgenic and WT poplars was performed using quantitative reverse-transcription PCR with cDNA as a template. Three independent experiments were performed. Lane M, 2K molecular mass marker; lane 1, negative control (WT); lanes 2–9, transgenic lines 1–8, respectively. Significant differences are denoted by asterisks (*$P < 0.05$; **$P < 0.01$; ***$P < 0.001$).
