# Supplementary material for: Characterization, expression profiling, and functional analysis of a Populus trichocarpa defensin gene and its potential as an anti-Agrobacterium rooting medium additive
Source: Sci Rep. 2019 Oct 25;9:15359. doi: 10.1038/s41598-019-51762-0 (PMC6814764; doi:10.1038/s41598-019-51762-0)
Supplement: Supplementary file 5 — Supplementary 5 [file 41598_2019_51762_MOESM5_ESM.pdf]

Characterization, expression profiling, and functional analysis of a *Populus trichocarpa* defensin gene and its potential as an anti-*Agrobacterium* rooting medium additive

Hui Wei[1,a], Ali Movahedi[1,a], Chen Xu[1,2,a], Weibo Sun[1,a], Lingling Li[1], Dawei Li[1], Qiang Zhuge[1]*

[1]Co-Innovation Center for Sustainable Forestry in Southern China, Key Laboratory of Forest Genetics & Biotechnology, Ministry of Education, College of Biology and the Environment, Nanjing Forestry University. Nanjing 210037, China

[2]Jiangsu Provincial Key Construction Laboratory of Special Biomass Resource Utilization, Nanjing Xiaozhuang University, Nanjing, 211171, China

*Correspondence should be addressed to Qiang Zhuge: Co-Innovation Center for Sustainable Forestry in Southern China, Key Laboratory of Forest Genetics and Biotechnology, Ministry of Education, College of Biology and the Environment, Nanjing Forestry University, Nanjing 210037, China. E-mail: qzhuge@njfu.edu.cn; Fax: +86 25 85428701

[a] These authors are contributed equally as the first author

Hui Wei: 15850682752@163.com

Ali Movahedi: ali_movahedi@njfu.edu.cn

Chen Xu: xuchenidea@hotmail.com

Weibo Sun: cz851115@126.com

Lingling Li: 1162520689@qq.com

Dawei Li: dwli@njfu.edu.cn

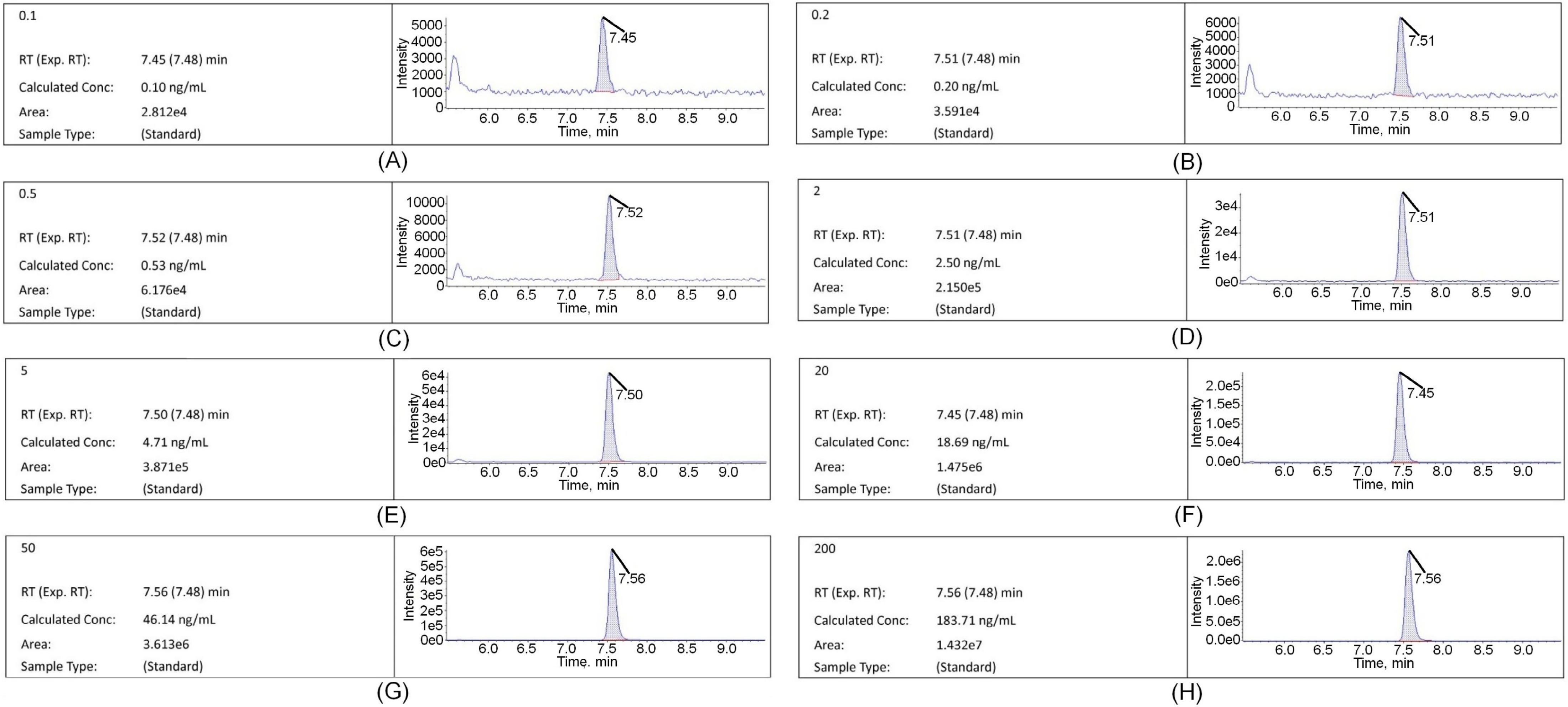

the name of material	calibration curve	correlation coefficient	weight
IAA	y = 7.78595e4 x + 20478.19669	0.99230	1/x2

(I)

Figure S5. HPLC-MS/MS chromatogram of IAA standards and equations for IAA. HPLC-MS/MS chromatogram of standard IAA at 0.1 (A), 0.2 (B), 0.5 (C), 2 (D), 5 (E), 20 (F), 50 (G), and 200 (H) ng/mL were dissolved in methanol/0.1% formic acid. (I) Equations for the IAA standard curves.
