# Supplementary material for: Characterization, expression profiling, and functional analysis of a Populus trichocarpa defensin gene and its potential as an anti-Agrobacterium rooting medium additive
Source: Sci Rep. 2019 Oct 25;9:15359. doi: 10.1038/s41598-019-51762-0 (PMC6814764; doi:10.1038/s41598-019-51762-0)
Supplement: Supplementary file 10 — Supplementary 10 [file 41598_2019_51762_MOESM10_ESM.pdf]

**Characterization, expression profiling, and functional analysis of a *Populus trichocarpa* defensin gene and its potential as an anti-*Agrobacterium* rooting medium additive**

Hui Wei[1,a], Ali Movahedi[1,a], Chen Xu[1,2,a], Weibo Sun[1,a], Lingling Li[1], Dawei Li[1], Qiang Zhuge[1]*

[1]Co-Innovation Center for Sustainable Forestry in Southern China, Key Laboratory of Forest Genetics & Biotechnology, Ministry of Education, College of Biology and the Environment, Nanjing Forestry University. Nanjing 210037, China

[2]Jiangsu Provincial Key Construction Laboratory of Special Biomass Resource Utilization, Nanjing Xiaozhuang University, Nanjing, 211171, China

*Correspondence should be addressed to Qiang Zhuge: Co-Innovation Center for Sustainable Forestry in Southern China, Key Laboratory of Forest Genetics and Biotechnology, Ministry of Education, College of Biology and the Environment, Nanjing Forestry University, Nanjing 210037, China. E-mail: qzhuge@njfu.edu.cn; Fax: +86 25 85428701

[a] These authors are contributed equally as the first author

Hui Wei: 15850682752@163.com
Ali Movahedi: ali_movahedi@njfu.edu.cn
Chen Xu: xuchenidea@hotmail.com
Weibo Sun: cz851115@126.com
Lingling Li: 1162520689@qq.com
Dawei Li: dwli@njfu.edu.cn

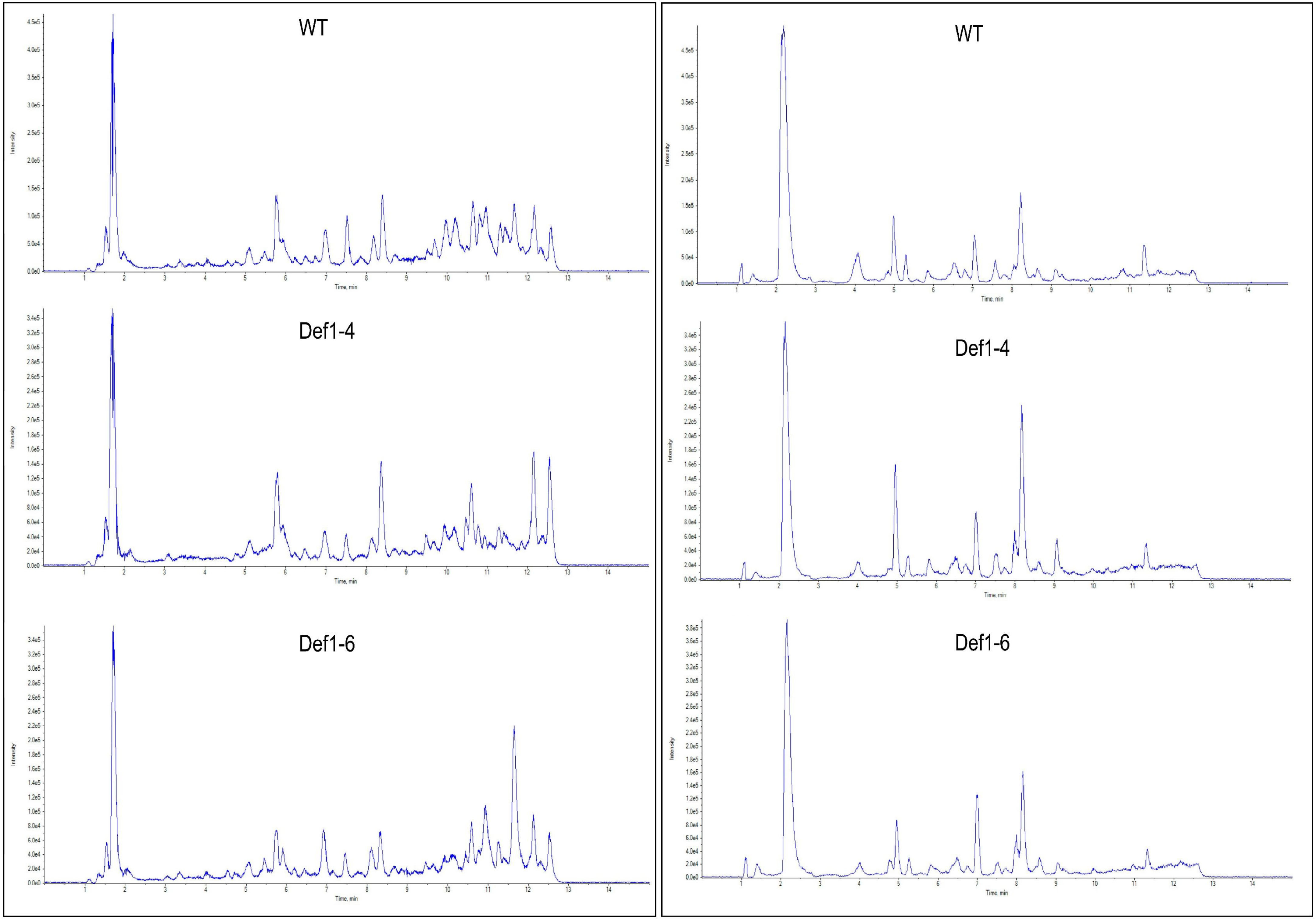
WT
Def1-4
Def1-6
Intensity
Time, min
WT
Def1-4
Def1-6
Intensity
Time, min

(A) (B)

Figure S10. High-performance liquid chromatography–tandem mass spectrometry image of raw data for transgenic and wild-type poplars treated with (A) auxin and (B) abscisic acid, gibberellin, jasmonic acid, and salicylic acid.
