# Supplementary material for: Characterization, expression profiling, and functional analysis of a Populus trichocarpa defensin gene and its potential as an anti-Agrobacterium rooting medium additive
Source: Sci Rep. 2019 Oct 25;9:15359. doi: 10.1038/s41598-019-51762-0 (PMC6814764; doi:10.1038/s41598-019-51762-0)
Supplement: Supplementary file 11 — Supplementary table [file 41598_2019_51762_MOESM11_ESM.pdf]

Characterization, expression profiling, and functional analysis of a *Populus trichocarpa* defensin gene and its potential as an anti-*Agrobacterium* rooting medium additive

Hui Wei[1,a], Ali Movahedi[1,a], Chen Xu[1,2,a], Weibo Sun[1,a], Lingling Li[1], Dawei Li[1], Qiang Zhuge[1]*

[1]Co-Innovation Center for Sustainable Forestry in Southern China, Key Laboratory of Forest Genetics & Biotechnology, Ministry of Education, College of Biology and the Environment, Nanjing Forestry University. Nanjing 210037, China

[2]Jiangsu Provincial Key Construction Laboratory of Special Biomass Resource Utilization, Nanjing Xiaozhuang University, Nanjing, 211171, China

*Correspondence should be addressed to Qiang Zhuge: Co-Innovation Center for Sustainable Forestry in Southern China, Key Laboratory of Forest Genetics and Biotechnology, Ministry of Education, College of Biology and the Environment, Nanjing Forestry University, Nanjing 210037, China. E-mail: qzhuge@njfu.edu.cn; Fax: +86 25 85428701

[a] These authors are contributed equally as the first author

Hui Wei: 15850682752@163.com
Ali Movahedi: ali_movahedi@njfu.edu.cn
Chen Xu: xuchenidea@hotmail.com
Weibo Sun: cz851115@126.com
Lingling Li: 1162520689@qq.com
Dawei Li: dwli@njfu.edu.cn

primer	Direction	Nucleotide sequence(5´-3´)
PtDef	Forward	ATGGAGATCAAGAGATCCTTTGG
PtDef	Reverse	TTAACAAAGC TTGGTGCAAA AAC
q-*PtDef*	Forward	TGCTCCTCATTGTCTTGGCT
q-*PtDef*	Reverse	GAACCCTTTGCATCTTCCCC
q-*Actin*	Forward	GCCATCTCTCATCGGAATGGAA
q-*Actin*	Reverse	AGGGCAGTGATTTCCTTGCTCA
PET-32a-*PtDef*	Forward	CGGGATCCATGGAGATCAAGAGATCCTTT
PET-32a-*PtDef*	Reverse	AAGGAAAAAAGCGGCCGCTTAACAAAGCTTGGTGCAAA
35S promoter	Forward	CTATCCTTCGCAAGACCCTTC
q-*AOC* (XP_002305951.2)	Forward	TGCGTGCTGATCCAAAACAA
q-*AOC* (XP_002305951.2)	Reverse	TGAAAGGGAACACAAGCTGC
q-*AOC* (XP_024461721.1)	Forward	TGTGCCTTTCTCCAACAAGC
q-*AOC* (XP_024461721.1)	Reverse	AAAGATCCCAGACCCACCAG
q-*LOX* (XP_002319014.3)	Forward	AGCACCCCTAACGAGTTTGA
q-*LOX* (XP_002319014.3)	Reverse	ACTCCTGCCAGCATTTCTCT
q-*LOX* (XM_002314512.3)	Forward	GGAGGGATAGATGTGAGGGC
q-*LOX* (XM_002314512.3)	Reverse	TGCCCTTACATCTCCCTG
q-*HPL* (XM_002321100.3)	Forward	GTCTTGAAACGCAGCTCCAA
q-*HPL* (XM_002321100.3)	Reverse	AAGCCACTTGTCCAGCATTG
q-*ICS* (GQ260071.1)	Forward	TTACGTCTCCAGGTGCCAAT
q-*ICS* (GQ260071.1)	Reverse	CTGAAAAGGACAGCAGAGCC
q-*PAL* (XM_002322848.3)	Forward	CCTCCAAGGTTACTCTGGCA
q-*PAL* (XM_002322848.3)	Reverse	TGCCAGTCAGTAATCCAGCA
q-*PR* (XM_006379094.2)	Forward	TTATGCTAACCAACGTGCCG
q-*PR* (XM_006379094.2)	Reverse	ATCCTAGGCGAGCAGAGTTC
q-*PR* (XM_024582168.1)	Forward	CCAAGACTCCCCACAAGACT
q-*PR* (XM_024582168.1)	Reverse	ACCCACAATTTCACCGCATC
q-*PR* (XM_002324451.3)	Forward	ACCGCTTCTTGGCTCCTCAA
q-*PR* (XM_002324451.3)	Reverse	AGTCCAGTCACTGCCACTAC
q-*PR* (XM_002319041.3)	Forward	GGTCTAGTGGTAGTGGTGGC
q-*PR* (XM_002319041.3)	Reverse	ATTGGTCACCCTCAAGCACT
q-*PR* (XM_006386317.2)	Forward	TGATGGAGTGTGCGAGGATT
q-*PR* (XM_006386317.2)	Reverse	CCGTCTTGCATGAACCACTT
q-*PR* (XM_002313896.3)	Forward	ACAACACGGTAGCAGCCTAT
q-*PR* (XM_002313896.3)	Reverse	CGAACTGAATTGCGCCAAAC
q-*APX* (XM_002312929.3)	Forward	GGAGTGAAGGCGAAACATCC
q-*APX* (XM_002312929.3)	Reverse	ATGACCTCCAGAAAGTGCCA
q-*CAT* (XM_002306391.3)	Forward	TGTCATTCACACTGGCAAGC
q-*CAT* (XM_002306391.3)	Reverse	CCAGAGACTTGTCAGCCTGA
q-*GST* (XM_006386691.2)	Forward	ACAGCTGCACAACGAGTTTT
q-*GST* (XM_006386691.2)	Reverse	TCTGCTTGCCTGGGATAACA
q-*SOD* (XP_006375790.1)	Forward	CCATGTTCATGCCCTTGGAG
q-*SOD* (XP_006375790.1)	Reverse	TTGAGACAGTAGCAGTGCCA
q-*RbohA* (XP_006368770.2)	Forward	CTTGGTGCCTTTGACGACA
q-*RbohA* (XP_006368770.2)	Reverse	TCCAGTCACACCTTCAGTCC
q-*RbohB* (XP_024456163.1)	Forward	GTGACTGGACATCGCAACTC
q-*RbohB* (XP_024456163.1)	Reverse	CACCAATGCCAAGTCCAACA
q-*LTP* (XM_002312093.3)	Forward	GATGGCTCCTTGTGCATCAG
q-*LTP* (XM_002312093.3)	Reverse	CCACAGGACGATCAGCAATG
q-*LTP* (XM_002314714.3)	Forward	TGCTGCTCCAAAGTTACTGC
q-*LTP* (XM_002314714.3)	Reverse	ATGGGAGCGTGTACTTTCCA
q-*LTP* (XM_002315155.3)	Forward	GGCTGGTGAATGTGGGAAAA
q-*LTP* (XM_002315155.3)	Reverse	CCACAGGACGATCAGCAATG
q-*LTP* (EF144767.1)	Forward	CCACCAACTAACCTATGCTGC
q-*LTP* (EF144767.1)	Reverse	TTAGCATTTGGGGAAGGGAGT
q-*LTP* (XM_002322032.3)	Forward	GGTGCTTCTTCTGGCTCAAG
q-*LTP* (XM_002322032.3)	Reverse	CCTGGCATTTGGTGTGTTGA

Table S1: Primer sequences used in this study.
